# Pregnenolone and progesterone production from natural sterols using recombinant strain of *Mycolicibacterium smegmatis* mc^2^ 155 expressing mammalian steroidogenesis system

**DOI:** 10.1186/s12934-024-02385-2

**Published:** 2024-04-09

**Authors:** Mikhail Karpov, Nicolai Strizhov, Ludmila Novikova, Tatyana Lobastova, Sergey Khomutov, Andrei Shutov, Alexey Kazantsev, Marina Donova

**Affiliations:** 1 G.K. Skryabin Institute of Biochemistry and Physiology of Microorganisms, RAS, Federal Research Center “Pushchino Scientific Center for Biological Research of the Russian Academy of Sciences”, Pushchino, 142290 Russia; 2 Belozersky Institute of Physico-Chemical Biology, Lomonosov Moscow State University, Leninskie Gory 1/40, Moscow, 119234 Russia; 3 Chemistry Department, Lomonosov Moscow State University, Leninskie Gory 1/3, Moscow, 119991 Russia

**Keywords:** Cytochrome P450scc, Progesterone, Pregnenolone, Sterols, 3-methoxymethylated steroids, *Mycolicibacterium smegmatis* mc^2^ 155

## Abstract

**Background:**

Pregnenolone and progesterone are the life-important steroid hormones regulating essential vital functions in mammals, and widely used in different fields of medicine. Microbiological production of these compounds from sterols is based on the use of recombinant strains expressing the enzyme system cholesterol hydroxylase/C20-C22 lyase (CH/L) of mammalian steroidogenesis. However, the efficiency of the known recombinant strains is still low. New recombinant strains and combination approaches are now needed to produce these steroid hormones.

**Results:**

Based on *Mycolicibacterium smegmatis*, a recombinant strain was created that expresses the steroidogenesis system (CYP11A1, adrenodoxin reductase, adrenodoxin) of the bovine adrenal cortex. The recombinant strain transformed cholesterol and phytosterol to form progesterone among the metabolites. When 3-methoxymethyl ethers of sterols were applied as bioconversion substrates, the corresponding 3-ethers of pregnenolone and dehydroepiandrosterone (DHEA) were identified as major metabolites. Under optimized conditions, the recombinant strain produced 85.2 ± 4.7 mol % 3-methoxymethyl-pregnenolone within 48 h, while production of 3-substituted DHEA was not detected. After the 3-methoxymethyl function was deprotected by acid hydrolysis, crystalline pregnenolone was isolated in high purity (over 98%, w/w). The structures of steroids were confirmed using TLC, HPLC, MS and ^1^H- and ^13^C-NMR analyses.

**Conclusion:**

The use of mycolicybacteria as a microbial platform for the expression of systems at the initial stage of mammalian steroidogenesis ensures the production of valuable steroid hormones—progesterone and pregnenolone from cholesterol. Selective production of pregnenolone from cholesterol is ensured by the use of 3-substituted cholesterol as a substrate and optimization of the conditions for its bioconversion. The results open the prospects for the generation of the new microbial biocatalysts capable of effectively producing value-added steroid hormones.

**Supplementary Information:**

The online version contains supplementary material available at 10.1186/s12934-024-02385-2.

## Introduction

Steroids are a superfamily of terpenoid lipids with a gonane core consisting of three cycloalkane and one cyclopentane rings. These compounds perform vital functions in all living organisms, they can regulate vital processes in vertebrates, acting as signaling molecules in signal transduction pathways by binding to the corresponding intracellular receptors [1–3].

**Fig. 1:**
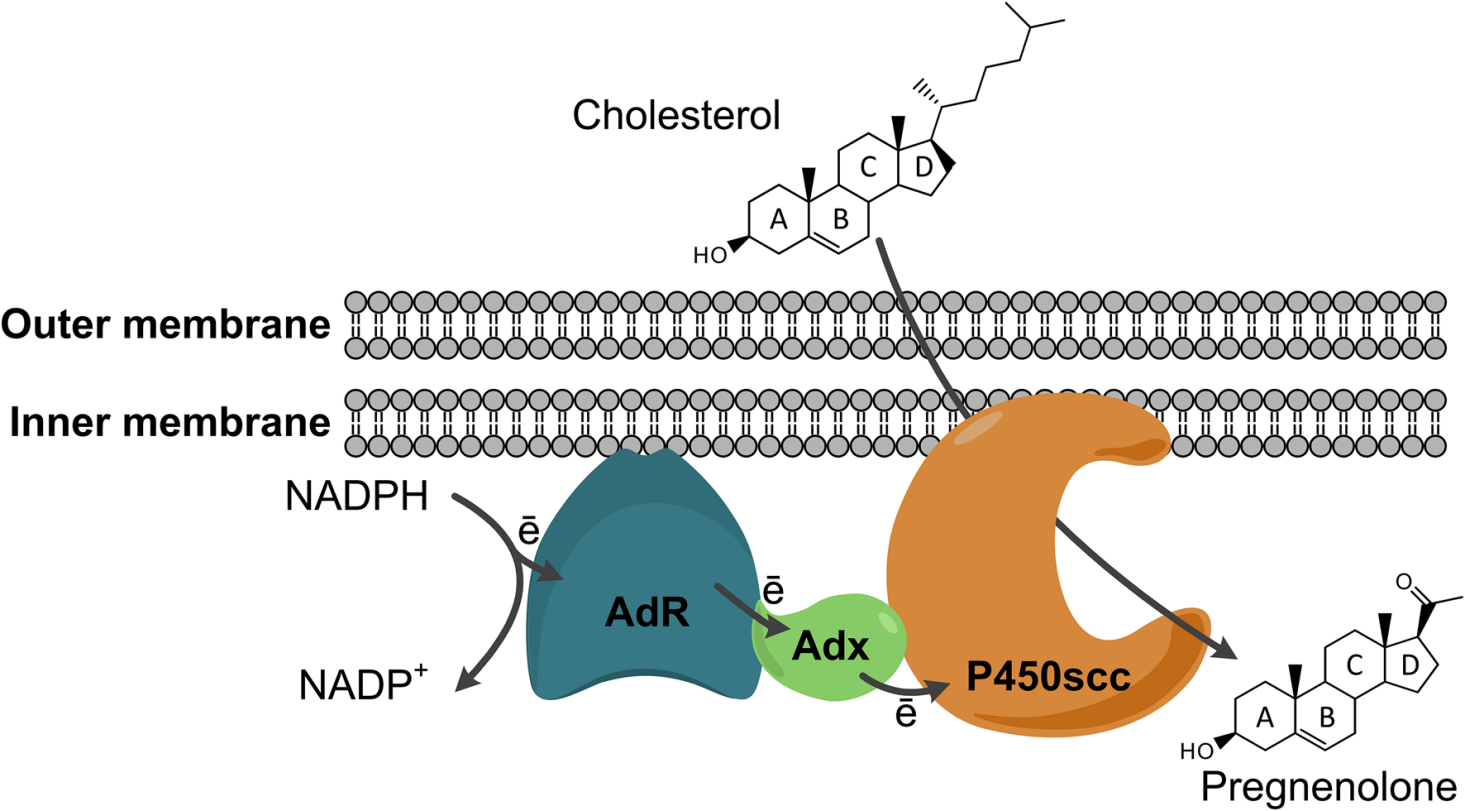
Schematic representation of the general organization of the mammalian cholesterol hydroxylase/C20-C22 lyase system in adrenocortical mitochondria. Cytochrome P450scc catalyzes the reaction of the cleavage of cholesterol side chain to form pregnenolone

Pregnenolone (3β-hydroxypregn-5-en-20-one) and progesterone (4-pregnen-3,20-dione) occupy a special place among steroid hormones. In mammals, pregnenolone is formed from cholesterol by the cholesterol hydroxylase/C20-C22 lyase steroidogenesis (CH/L) in adrenocortical mitochondria, and it is an important precursor of mineralocorticoids, glucocorticoids, sex hormones (androgens and estrogens), and neuroactive steroids [3, 4]. The CH/L enzyme system is composed of cytochrome P450scc (CYP11A1) catalyzing the cleavage of the cholesterol side chain, and it's natural redox partners, – NADPH-dependent flavine reductase, adrenodoxin reductase (AdR) and adrenodoxin (Adx) that donate electrons to cytochrome CYP11A1 (P450scc) [5] (Fig. 1).

Progesterone is a key progestogenic sex hormone formed from pregnenolone by the action of 3β-hydroxysteroid dehydrogenase/isomerase. It affects female fertility, pregnancy and embryonic development, may decrease negative effects of menopause and various pathologies in gynecology, plays an important role in brain function as a neurosteroid and serves as a key intermediate metabolite in the synthesis of sex and corticosteroid hormones [1, 2, 6].

Steroid-based drug production involves a combined synthesis of microbiological and chemical technologies [7]. The main substrates for the production of steroid synthons are phytosterols, along with diosgenin and cholesterol. The advantage of using plant sterols is their low cost and the possibility of their large-scale production from renewable plant raw materials such as soybean oil and by-products of the wood pulp industry [8]. Chemical modification of sterols, in particular protection at the 3rd position with a methoxymethyl substituent, allows microbiological removal of the sterol side chain and keeping the steroid core intact [9, 10]. Unlike other 3-substituted sterols, such as 3-acetylated sterols [11], 3-methoxymethyl-substituted sterols do not undergo enzymatic hydrolysis, which prevents the unwanted formation of 3-keto derivatives such as cholestenone and phytostenones [9]. Recently, the efficient biotransformation of 3-methoxymethyl-substituted phytosterols using *Mycobacterium* sp. NRRL B-3683 to produce DHEA (3β-hydroxyandrost-5-en-17-one), a hormone widely involved in physiological regulation in humans and mammals, has been reported [10].

Currently, pregnenolone and progesterone are produced mainly chemically by some multistep syntheses [12]. Recently, chemical synthesis of progesterone has been reported from 22-hydroxy-23,24-bisnorchol-4-ene-3-one (HBC, also known as BA, or 20-HMP), which in turn can be obtained from phytosterols using engineered *Mycolicibacterium* strains [13].

In the last two to three decades, research related to the creation of microbial strains capable of producing steroid hormones has been actively developing, which opens up prospects for replacing multi-stage chemical syntheses with environmentally friendly biotechnologies. Using genetic engineering methods, a new generation of microbial biocatalysts has been created that carry out cascades of reactions of structural modification of (phyto)sterols with the formation of steroid hormones [14–17].

Steroidogenic responses in mammals are studied using model systems based on the expression of heterologous proteins in microorganisms in in vitro and in vivo experiments. For example, expression of the bovine adrenal cortex CH/L system was studied in the yeasts *Saccharomyces cerevisiae* [18] and *Yarrowia lipolytica* [17]. The design of the endogenous sterol synthesis in these yeasts with the introduction of the mammalian CH/L system made it possible to synthesize pregnenolone from the components of the nutrient medium, but the steroid yield did not exceed 60–78 mg/L [17, 18]. Co-expression of human 3β-hydroxysteroid dehydrogenase along with mammalian CH/L in *S. cerevisiae* resulted in the formation of progesterone [18].

A chimeric construct mCYP11A1-L-AdR (mutant CYP11A1 fused to AdR via a flexible linker), Adx and AdR-homologous yeast reductase Arh1 were co-expressed in *Mycobacterium neoaurum* (syn. *Mycolicibacterium neoaurum*). The combination of the recombinant *M. neoaurum* cells and InP nanoparticles for light regeneration of NADPH provided an increase in progesterone synthesis up to 235 ± 50 mg/L [19].

*Escherichia coli* strains are a convenient host for the expression of recombinant P450s due to the lack of their own cytochromes P450, while at the same time providing a high level of expression of heterologous P450s in their active forms [20]. However, conversion of cholesterol by the recombinant *E. coli* strain with cytochrome P450scc and its redox partners resulted in low yield of pregnenolone (0.42–0.62 mg/L) [14, 21]. Expression of CYP11A1 in combination with essential electron transport proteins in the gram-positive soil bacterium *Bacillus megaterium* allowed the production of pregnenolone from cholesterol and β-sitosterol, and 7-dehydropregnenolone from 7-dehydrocholesterol. The yield of products was 25–95 mg/L depending on the substrate used [16].

The widespread non-pathogenic strains of *Mycolicibacterium smegmatis* are also being used for genetic manipulation to create recombinant strains with beneficial properties. Thus, the production of 14α-hydroxy derivatives of C19 steroids from sterols in one fermentation step became possible after the constructed synthetic operon containing CYP103168 and its natural partner CPR64795 from *C. lunatus* was transferred to *M. smegmatis* [22].

In this study, a recombinant strain of *M. smegmatis* mc^2^ 155 was created, additionally carrying in its cells a plasmid genetic construct encoding the enzyme system cholesterol hydroxylase/C20-C22 lyase of steroidogenesis of the bovine adrenal cortex (P450scc/Adx/AdR) (pNS11). Its activity was studied in vivo for the production of progesterone from sterols (cholesterol, phytosterol) and 3-methoxymethylpregnenolone (MOM-pregnenolone) from substituted sterols (3-methoxymethylcholesterol (MOM-cholesterol), 3-methoxymethylphytosterol (MOM-phytosterol) with subsequent removal of 3-methoxymethyl protection and regeneration of 3β-hydroxyl group to form pregnenolone.

## Results

### Creation of recombinant ***M. smegmatis*** mc^2^ 155 strain and estimation of its activity towards sterols and their 3-methoxymethyl derivatives

**Fig. 2:**
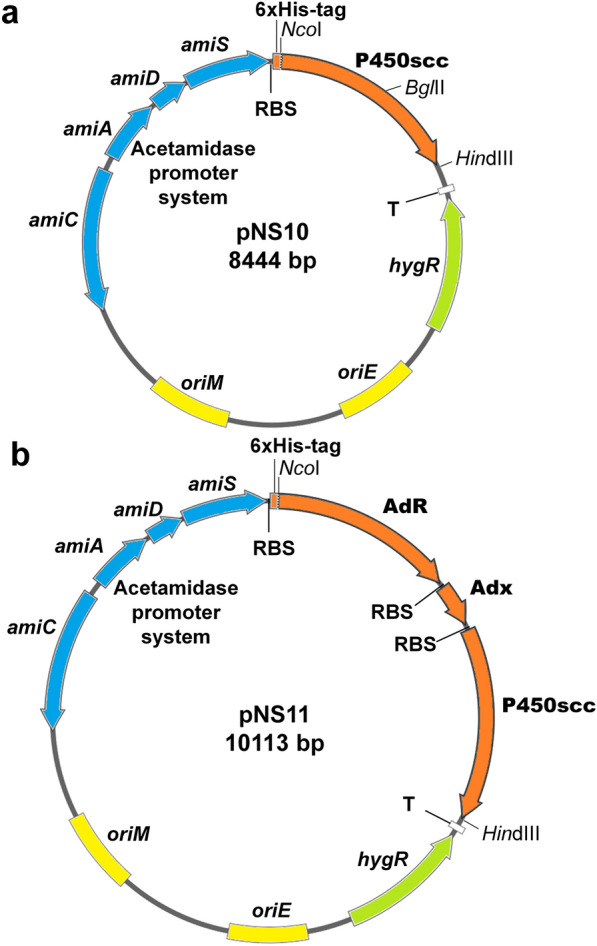
Structures of the plasmids for gene expression in mycolicibacteria. **a** The plasmid pNS10 contains cDNA encoding bovine cytochrome P450scc. **b** The plasmid pNS11 contains cDNAs for bovine AdR, Adx (1–108 aa) and cytochrome P450scc in a single expression cassette driven by acetamidase promoter system (*amiC*, *amiA*, *amiD* and *amiS*). The plasmids include respective origins of replication (*oriE* and *oriM*) for propagation in *E. coli* and *M. smegmatis*, transcription terminator T1 from the *E. coli rrn*B gene (T), and hygromycin B resistance marker (*hygR*). *Nco*I, *Hin*dIII and *Bgl*II restriction sites used at cloning procedure are designated

The plasmids pNS10 and pNS11 were constructed using the shuttle vector pMyNT (Fig. 2). The resulting plasmid pNS10 contains cDNA copy of mature P450scc protein gene from bovine adrenal cortex under the control of mycobacterial inducible acetamidase promoter (Fig. 2a). cDNA copies of the bovine *AdR, Adx* and *CYP11A1* genes encoding mature forms of AdR, Adx (1–108 aa) and cytochrome P450 cholesterol hydroxylase/C20-C22 lyase (P450scc) were combined with acetamidase promoter in the pNS11 construct generating an artificial tricystronic operon (Fig. 2b). The cDNA sequences encoding P450scc in pNS10 and AdR in pNS11 were fused in frame with the region encoding the polyhistidine tag (Fig. 2).

The vector pMyNT, as well as the plasmids pNS10 and pNS11 were transferred into *M. smegmatis* mc^2^ 155 cells. Induction with acetamide resulted in a high level of expression of heterologous genes in mycolicibacteria. Analysis of protein synthesis (SDS-PAGE and Western immunoblotting) showed that recombinant mycolicibacteria synthesize proteins corresponding in molecular weights to cytochrome P450scc proteins (58.8 kDa in cells with pNS10, 56.6 kDa in cells with pNS11), AdR (52.9 kDa) and Adx (12.2 kDa), which are immunospecifically detected by antibodies raised against bovine proteins P450scc, AdR and Adx, respectively (Additional file 1: Fig. S1 and S2).

The obtained recombinant strain of *M. smegmatis* mc^2^ 155 with plasmid pNS11 was tested for the ability to cleave the the bond between C20 and C22 of the side chain of sterols (cholesterol, phytosterol) and their 3-methoxymethyl derivatives (MOM-cholesterol, MOM-phytosterol).

**Fig. 3:**
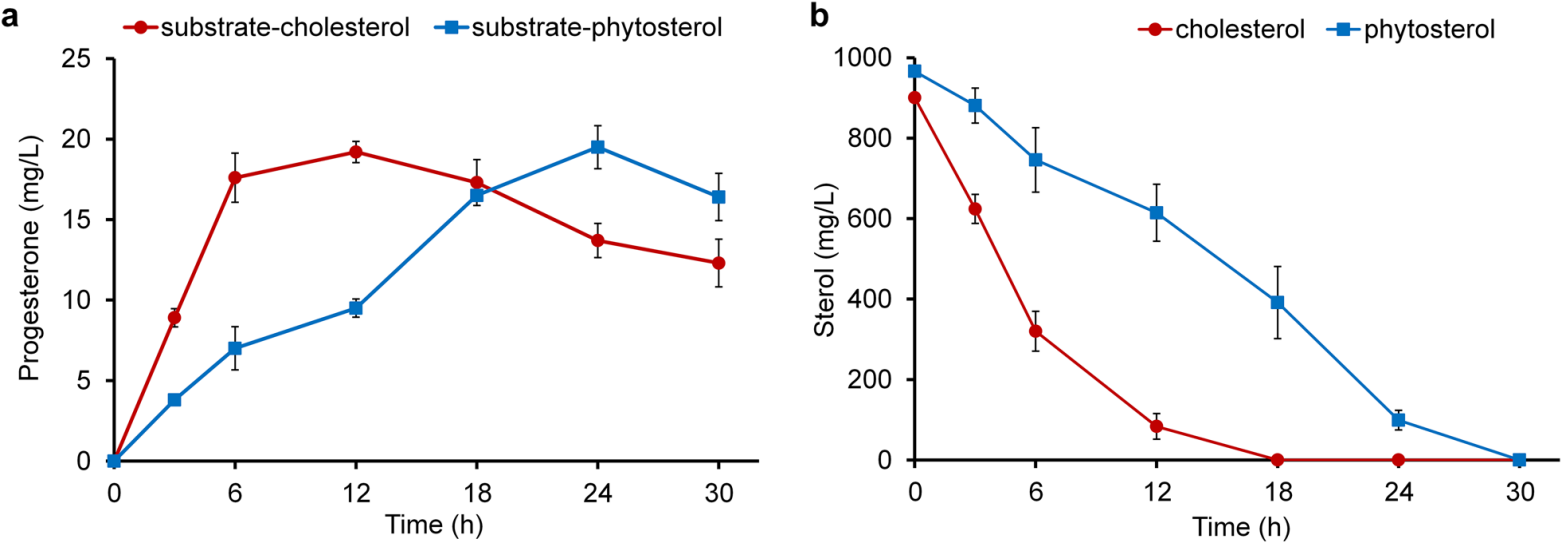
The conversion of sterols (2.33 mM) by the recombinant *M. smegmatis* mc^2^ 155 pNS11 strain. **a** Curves of progesterone accumulation from cholesterol and phytosterol, **b** cholesterol and phytosterol utilization

The conversion of cholesterol and phytosterol by the recombinant strain *M. smegmatis* mc^2^ 155 pNS11 led to the accumulation of progesterone (Fig. 3a), whose structure was confirmed by MS data (Additional file 1: Fig. S3 and S4).

Progesterone reached maximum level of 19.2 ± 0.66 mg/L within 12 h of cholesterol bioconversion (Fig. 3a), while full cholesterol depletion was observed within 18 h (Fig. 3b). The rate of phytosterol to progesterone conversion was inferior to that of cholesterol, and the maximum yield of the product reached 19.5 ± 1.33 mg/L at 24 h of transformation (Fig. 3a). The time of complete conversion of phytosterol by the strain also increased up to 30 h of transformation (Fig. 3b).

To avoid the oxidation of 3β-hydroxyl group, ∆^5^ → ∆^4^-isomerization and, accordingly, the loss of the center of chirality at C3, the structure of the 3β-hydroxy-5-ene sterol core was protected chemically. In particular, 3-methoxymethylated derivatives of cholesterol and phytosterol were synthesized and used as bioconversion substrates. Methoxymethyl group is one of the most powerful protective agents for the hydroxyl group. Methoxymethyl ether is stable in both basic and weakly acidic environments, which distinguishes it favorably from silyl, alkoxyacyl, or benzyl derivatives [23].

The methoxymethylated protection is resistant to esterase activity, and upon completion of the 3β-hydroxy-5-ene bioconversion, the steroid structure of the product can be easily regenerated by acid hydrolysis (deprotected with regeneration of 3β-hydroxyl) to form pregnenolone.

**Fig. 4:**
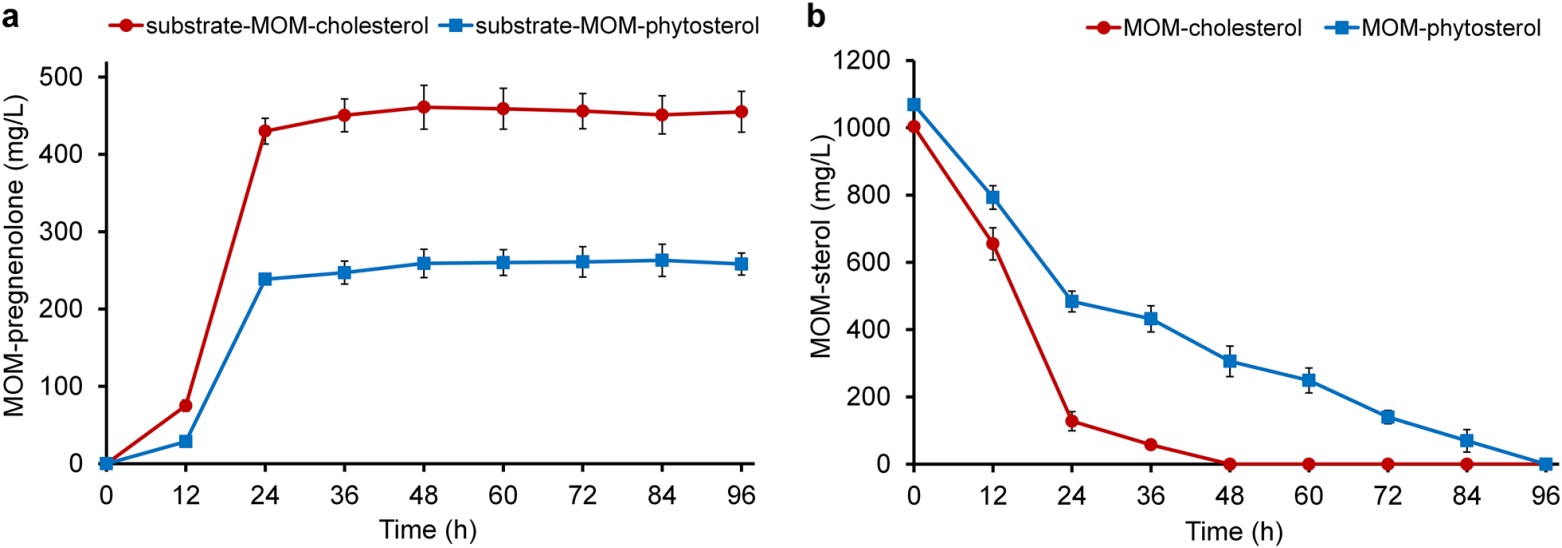
The conversion of MOM-sterols (2.33 mM) by the recombinant *M. smegmatis* mc^2^ 155 pNS11 strain. **a** Curves of MOM-pregnenolone accumulation from MOM-cholesterol and MOM-phytosterol, **b** MOM-cholesterol and MOM-phytosterol utilization

The recombinant strain *M. smegmatis* mc^2^ 155 pNS11 was able to transform sterols (cholesterol, phytosterol) protected at C3 by the methoxymethyl group. The transformation of MOM-cholesterol (2.33 mM) resulted in a high yield of MOM-pregnenolone (461 ± 28.3 mg/L) within 48 h (Fig. 4a). The yield of MOM-pregnenolone from MOM-phytosterol was significantly lower and reached to 263 ± 20.9 mg/L within 84 h (Fig. 4a). The rate of the 3-substituted substrates depletion also differed. MOM-cholesterol is completely transformed by *M. smegmatis* mc^2^ 155 pNS11 within 48 h, and MOM-phytosterol − within 96 h (Fig. 4b).

Thus, the rate of transformation of the studied substrates (cholesterol, phytosterol, MOM-cholesterol, MOM-phytosterol) by the recombinant *M. smegmatis* mc^2^ 155 pNS11 strain varied. Of the four tested substrates, cholesterol was the fastest to transform, and MOM-phytosterol was the slowest. The yield of MOM-pregnenolone was also highest and amounted to 54.9 mol % when using MOM-cholesterol as a substrate.

### Optimization of acetamide induction conditions

**Fig. 5:**
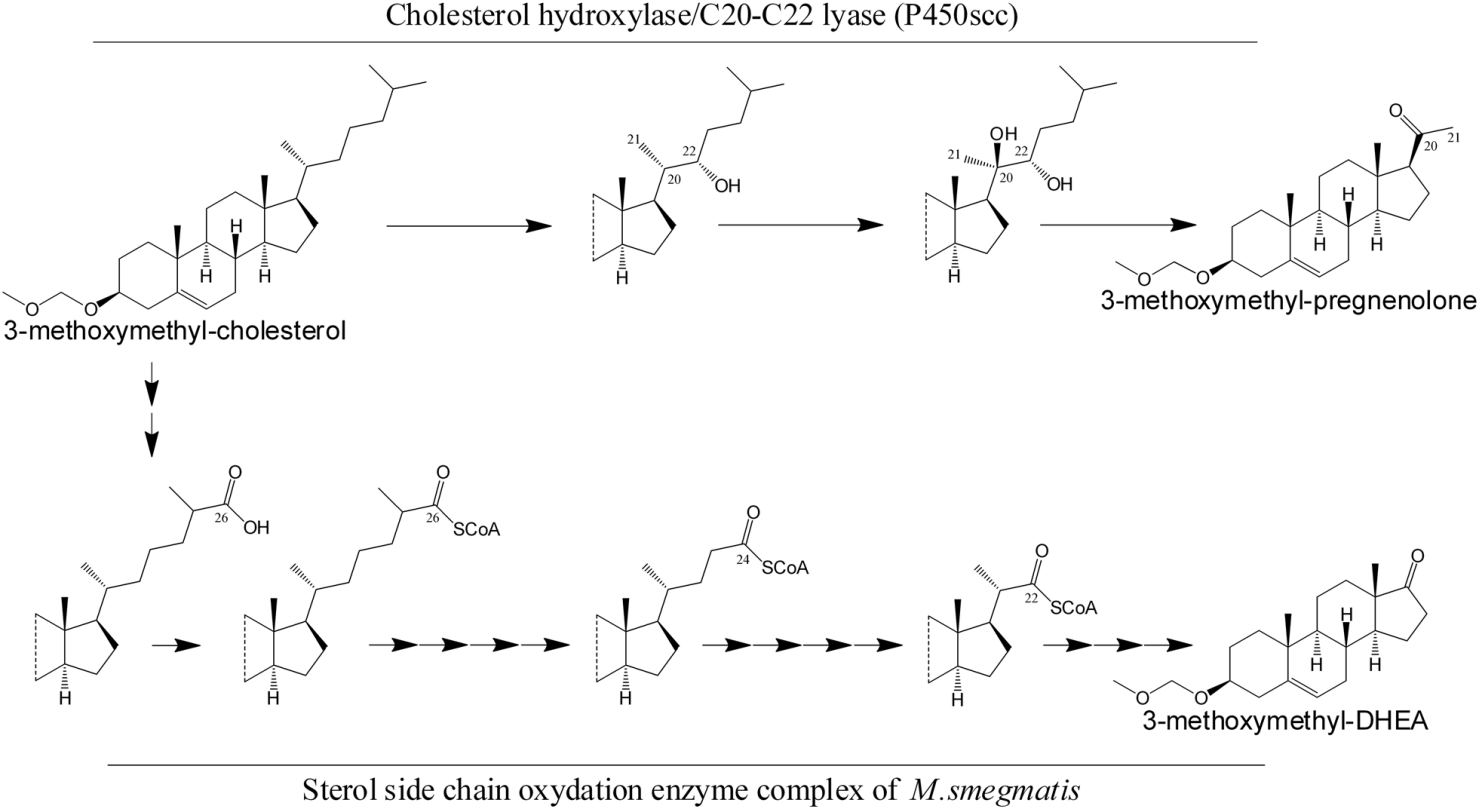
MOM-Cholesterol bioconversion by *M. smegmatis* mc^2^ 155 pNS11 to form MOM-DHEA and MOM-pregnenolone

The effect of the duration and additions of the inducer, acetamide, on the activity of CH/L in *M. smegmatis* mc^2^ 155 pNS11 was studied using MOM-cholesterol as a substrate. The gene expression of CH/L in *M. smegmatis* mc^2^ 155 pNS11 resulted in the cleavage of the carbon fragment of the side chain of the 3-substituted sterol with the formation MOM-pregnenolone. At the same time, these reactions compete with the side chain degradation by own enzymes in *M. smegmatis* mc^2^ 155 pNS11 to form the MOM-substituted C17-ketosteroid identified as MOM-DHEA (Fig. 5).

**Fig. 6:**
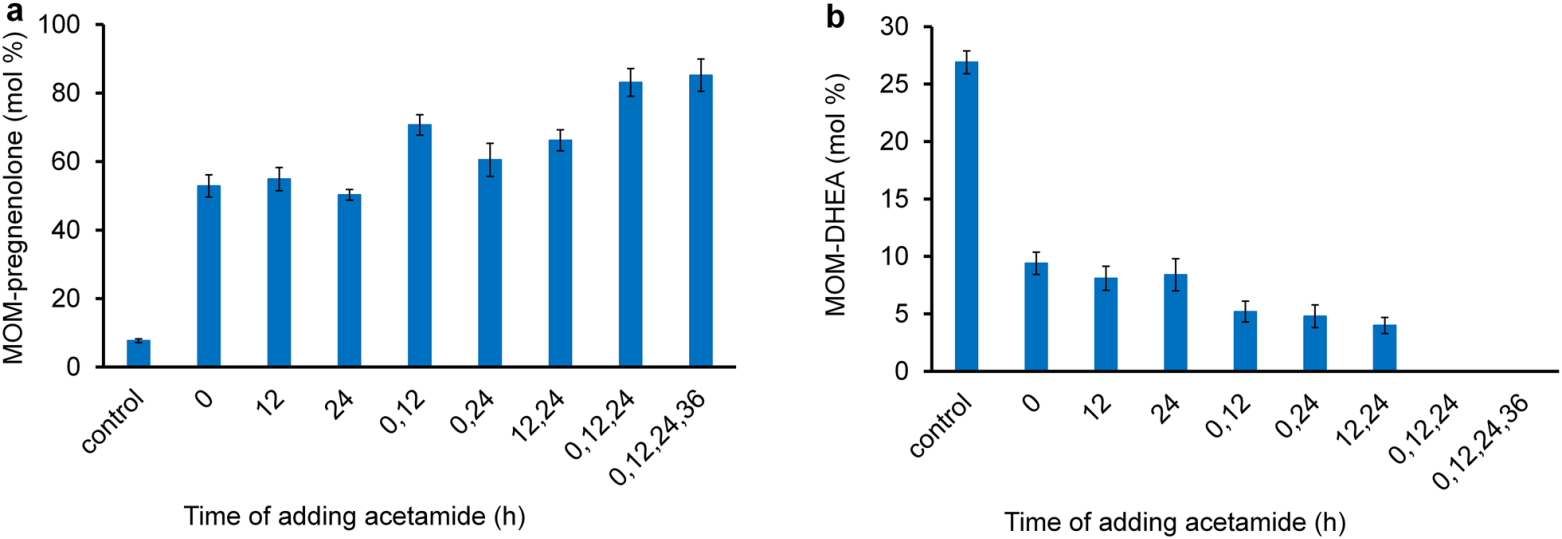
The influence of adding acetamide on the bioconversion of MOM-cholesterol by *M. smegmatis* mc^2^ 155 pNS11. **a** The yield of MOM-pregnenolone, **b** the yield of MOM-DHEA. Control—without adding acetamide; the single application of acetamide: at 0, 12 or 24 h; the double application of acetamide: at 0 and 12 h; 12 and 24 h or 0 and 24 h; the triple application of acetamide at 0, 12 and 24 h; the quadruple addition—at 0, 12, 24 and 36 h after the strain inoculation. The biotransformation time—48 h; final acetamide concentration in the medium—2 g/L

With a single addition of acetamide to the medium at 0, 12 or 24 h of strain growth, the yield of MOM-pregnenolone increased by 6.5–7.1 times compared to the control variant (without induction) and reached 50.3–54.9 ± 1.6–3.4 mol % within 48 h of MOM-cholesterol transformation (Fig. 6a).

Double addition of acetamide during strain growth ensured a further increase in the MOM-pregnenolone content to 60.5–70.7 ± 3.0–4.8 mol % over 48-h conversion (Fig. 6a). The time of adding acetamide influenced on the accumulation of the target product. When the inducer was added twice at 0 and 12 h of growth, maximum yield of MOM-pregnenolone was observed (Fig. 6a). A further increase in MOM-pregnenolone accumulation was observed when acetamide was added three times (0, 12 and 24 h) and four times (0, 12, 24 and 36 h) to reach a maximum value of 83.1–85.2 ± 4.0–4.7 mol % after 48 h of transformation, this was 10.8–11.1 times higher than in the control variant (Fig. 6a).

The accumulation of the by-product, MOM-DHEA, was highest in the control (in the absence of acetamide) and amounted to 26.9 ± 0.99 mol % within 48 h of MOM-cholesterol bioconversion (Fig. 6b). Single inducer addition resulted in lower yield of MOM-DHEA (8.1–9.4 ± 0.97–1.04 mol %) (Fig. 6b). Further decrease in MOM-DHEA accumulation (to 4.0–5.2 ± 0.7–0.9 mol %) was observed when using double addition of acetamide (Fig. 6b). MOM-DHEA was not detected among the metabolites with triple or quadruple addition of acetamide (Fig. 6b).

Thus, multiple additions of acetamide to the bioconversion medium simultaneously with the strain inoculation and additionally every 12 h provided high expression of the genes coding for mammalian CH/L in the *M. smegmatis* mc^2^ 155 pNS11 strain. As a result, the yield of MOM-pregnenolone reached more than 85% mol, while undesirable accumulation of MOM-DHEA was not observed.

### Cleavage of methoxymethyl protecting group

**Fig. 7:**
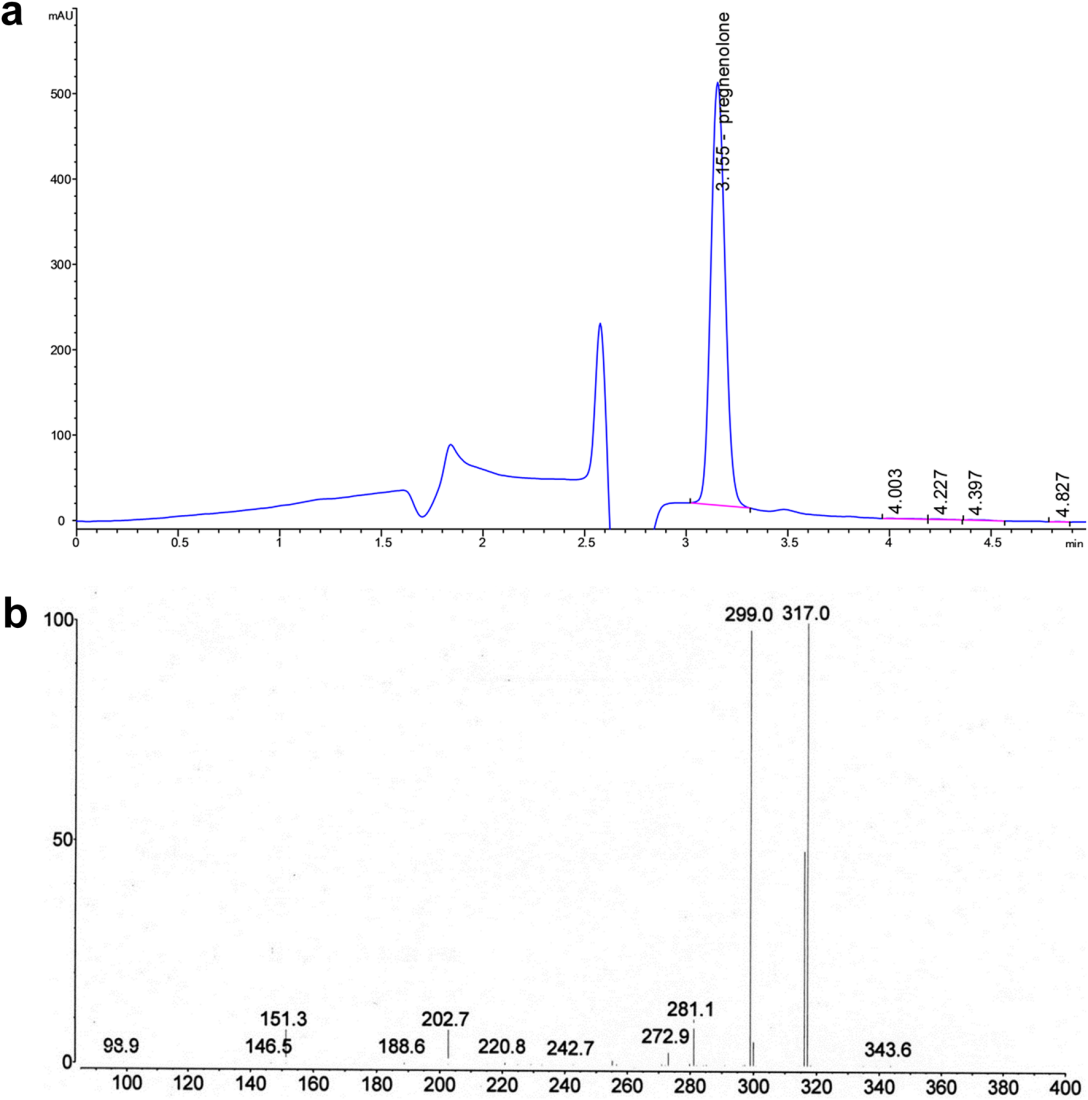
HPLC and mass analysis of crystalline pregnenolone obtained after acid hydrolysis of MOM-pregnenolone. **a** HPLC profile; **b** mass spectrum

MOM-pregnenolone, obtained as a result of the bioconversion of MOM-cholesterol (1 g/L) by the recombinant strain *M. smegmatis* mc^2^ 155 pNS11, was subjected to acid hydrolysis to regenerate the hydroxyl group at C3. Removal of methoxymethyl protection should be carried out under mild reaction conditions to avoid racemization of the stereocenter at C3 [24, 25]. We used an efficient streamlined procedure for selective deprotection of methoxymethyl ether in acidic water-organic media. The method provided almost quantitative regeneration of the 3-hydroxy group of pregnenolone (see “Materials and Methods”). Total content of steroid impurities did not exceed 1% (w/w). Residual MOM-pregnenolone was at trace levels. The yield of crystalline pregnenolone with a purity of 98.6% (w/w) (Fig. 7a) was 420 mg from 1 L of biotransformation medium. The structure of pregnenolone was confirmed by MS (C_21_H_32_O_2_ [M + H^+^] = 317.0) (Fig. 7b) and ^1^H- and ^13^C-NMR analyses (Additional file 1: Fig. S5 and S6). Spectral data of the product were the following for ^1^H-NMR (400 MHz, CDCl_3_) δ: 5.35 (m, 1H, H-6), 3.53 (m, 1H, H-3α), 2.54 (m, 1H, H-17α), 2.13 (s, 3H, H-21), 1.01 (s, 3H, H-19), 0.63 (s, 3H, H-18) and ^13^C-NMR (100.6 MHz, CDCl_3_) δ: 209.6, 140.7, 121.3, 71.6, 63.7, 56.9, 49.9, 44.0, 42.2, 38.8, 37.2, 36.5, 31.8, 31.7, 31.5, 31.5, 24.4, 22.8, 21.0, 19.3, 13.2.

## Discussion

The construction of plasmids with functional monooxygenase systems for co-expression of proteins in bacterial cells has been described in several papers [14, 16, 19, 21]. For co-expression of the protein components of the CH/L system, several polycistronic plasmids based on pBar and pTrc99A were constructed [14, 21]. The use of cDNAs encoding the bovine P450scc, Adx, AdR proteins and RBS sites before each of the heterologous cDNA in one transcription unit results in independent translation of the individual proteins. Co-expression of all components of the CH/L system in *E. coli* DH5αc cells was controlled by IPTG. However, the efficiency of biotransformation of cholesterol into pregnenolone by cells transformed with these plasmids was low [14, 21].

Unlike *E. coli*, *M. smegmatis* mc^2^ 155 possesses an efficient transport system for exogenous steroid compounds, has a high transformation rate, and is convenient for the expression of foreign genes of steroidogenesis [2, 26]. Previously, as a recipient host, *M. smegmatis* mc^2^ 155 was used to create a recombinant strain for the production of testosterone from cholesterol [27] and 14α-hydroxy derivatives from AD and ADD [22].

The activity of recombinant P450 cytochromes depended on the recipient strain used and the presence or absence of suitable redox partners proteins in host cells [14, 16, 28]. For the manifestation of the activity of bacillary CYP106A1 and CYP106A2 in *M. smegmatis* mc^2^ 155 cells, the host’s own proteins were sufficient to ensure electron transport to recombinant cytochromes P450 [28]. However, to ensure the synthesis of pregnenolone in recombinant cells of *Saccharomyces cerevisiae* [18], *E. coli* [14, 21] and *Bacillus megaterium* [16] the presence of all the components of the mammalian CH/L (P450scc/Adx/AdR) is required.

The need for foreign electron carriers AdR and Adx in the P450scc/Adx/AdR system was proven on a recombinant *E. coli* strain expressing a single *CYP11A1* gene, which showed the absence of the target product (pregnenolone) in cholesterol bioconversion [14].

In this work, the construction of a tricistronic co-expressing vector (pNS11) under an acetamidase promoter and its transformation in *M. smegmatis* mc^2^ 155 cells made it possible to create a recombinant strain capable of converting sterols (cholesterol, phytosterol) and their 3-substituted derivatives. Due to the complex regulon the use of the acetamidase promoter allows chemoinduction with acetamide, which leads to overexpression of transferred genes in mycobacteria and allows the simultaneous expression of several genes combined into a polycistronic matrix [29].

In bioconversions, the use of sterols with different structures affected the time of complete transformation of substrates by the *M. smegmatis* mc^2^ 155 pNS11 strain. The lengthening of the side chain, in the case of phytosterol, led to an increase in the time of complete conversion of the substrate compared to cholesterol. The results correlate with experimental data, where it was shown, that CYP11A1 converted plant sterols (β-sitosterol, campesterol) to pregnenolone in vitro with less efficiency compared to cholesterol [30, 31]. The presence of a methoxymethyl substituent at C3 in cholesterol and phytosterol reduced the rate of substrate transformation possibly because the MOM-derived sterols are even much more hydrophobic as compared with natural sterols. Previously, the effects of the influence of the structure of the side chain and the presence of the substituent at the 3rd position in sterols on their microbial conversion were studied [9, 32]. In the series of sterols, conversion time increased in the following order: cholesterol, campesterol, β-sitosterol, stigmasterol, dehydrocholesterol and ergosterol [32]. For example, the presence of an acetate substituent at the 3rd position in ergosterol during its conversion by *Mycobacterium* sp. VKM Ac-1815D resulted in an increase in transformation time compared to the unsubstituted steroid [9].

It is known that the strain *M. smegmatis* mc^2^ 155 contains in its genome a complete set of genes involved in sterol catabolism. Sterol degradation can begin with oxidation of the C17 alkyl side chain or modification of the A ring of the steroid core. These two processes proceed independently of each other [9, 33]. In most cases the degradation of sterols by actinobacteria begins with the modification of the ring A including the oxidation of the 3β-hydroxy group and Δ^5^ → Δ^4^-isomerization of double bond [34]. In *M. smegmatis* mc^2^ 155 this reaction is catalyzed by two enzymes: cholesterol oxidase and 3β-hydroxysteroid dehydrogenase [35].

The synthesis of progesterone from sterols by the recombinant strain *M. smegmatis* mc^2^ 155 pNS11 is due to the presence of 3β-hydroxysteroid dehydrogenase or cholesterol oxidase, which transforms the resulting pregnenolone into a 3-keto-4-ene steroid, followed by its degradation under the action of sterol catabolism enzymes. Protection of the 3β-hydroxyl group of cholesterol and phytosterol with a methoxymethyl substituent made it possible to preserve the 5-ene configuration of the ring A in the steroid core. The main product of the bioconversion of MOM-cholesterol and MOM-phytosterol was MOM-pregnenolone. The inaccessibility of the ring A for the enzymes involved in the degradation of rings A and B made it possible to increase the selectivity of the process for obtaining MOM-pregnenolone. Noteworthy, it also prevents the formation of cholestenone from cholesterol which is not a suitable substrate for cytochrome P450scc (CYP11A1) [36].

At the same time, the side chain at C17 in the substituted sterols remained open to attack by the intrinsic enzymes of *M. smegmatis* involved in the degradation of the alkyl side chain, thus resulting in the accumulation of a by-product—MOM-DHEA. Increasing of the activity of the mammalian CH/L system in the recombinant strain in the presence of acetamide inducer resulted in the decrease of MOM-DHEA formation. This effect was enhanced by repeated addition of the inducer to the medium, which contributed to the complete suppression of the formation of MOM-DHEA and significant raising the yield of MOM-pregnenolone. In earlier published papers, the activation of the promoter and the expression of all components of the CH/L system in host cells of *E.coli* and *B. megaterium* was carried out by the inducer once [14, 16, 21]. Probably, a single introduction of the inducer into the medium did not allow achieving the maximum co-expression of proteins (P450scc, Adx, and AdR). As a result, a low yield of pregnenolone was observed during cholesterol bioconversion by these strains.

Microbiological preparation of MOM-pregnenolone from MOM-cholesterol with a high yield of more than 85% (mol) allowed us to isolate the target product in preparative amounts. An efficient method for removing the methoxymethyl group from a pregnenolone derivative produced crystalline pregnenolone with a yield of over 400 mg from 1 L of biotransformation medium.

The proposed pathway of the synthesis of pregnenolone is an example of a successful combination of various methods: the creation of a recombinant strain by genetic engineering methods, and application of chemical protection/deprotection of the functional groups at the microbial transformation.

## Conclusion

The cholesterol hydroxylase/C20-C22 lyase enzyme system catalyzing the initial step of mammalian steroidogenesis (CH/L) was implemented in *M. smegmatis* mc^2^ 155. The obtained recombinant strain *M. smegmatis* mc^2^ 155 pNS11 under the growth conditions selectively cleaves off the carbon fragment of the aliphatic side chain of sterols and their 3-methoxymethyl derivatives to form progesterone and MOM-pregnenolone, respectively. The highest yield of the target product—MOM-pregnenolone (85.2 ± 4.7 mol %) was obtained by bioconversion of MOM-cholesterol (1 g/L). The removal of chemical protection at the C3 position of MOM-pregnenolone resulted in the production of crystalline pregnenolone of high purity.

These data demonstrate the effectiveness of method for obtaining pregnenolone using a recombinant strain of *M. smegmatis* mc^2^ 155 pNS11 carrying a tricistronic vector with bovine *P450scc* (*CYP11A1*), *Adx* and *AdR* genes. To the best of our knowledge, this is a first report demonstrating pregnenolone production from MOM-(phyto)sterols using a whole-cell microbial system.

The results open up prospects for the application of engineered mycolicibacteria for biotechnological production of value-added steroid hormones from sterols which could be alternative to multistep and hazardous chemical syntheses.

## Materials and methods

### Materials

Cholesterol (cholest-5-en-3β-ol) was obtained from AppliChem (Germany); phytosterol (total sterols content—95.47%: β-sitosterol—42.39%, stigmasterol—26.08%, campesterol—23.48%, brassicasterol—3.52%)—from Jiangsu Spring Fruit Biological Products Co., Ltd. (China); pregn-4-ene-3,20-dione (progesterone); 3β-hydroxypregn-5-en-20-one (pregnenolone); 3β-hydroxyandrost-5-en-17-one (dehydroepiandrosterone, DHEA); acetamide—from Sigma-Aldrich (USA); yeast extract, bacto-peptone—from Difco (USA); randomly methylated β-cyclodextrin (MCD)—from Wacker-Chemie GmbH (Germany). Hygromycin B and DNA modifying enzymes were obtained from Thermo Fisher Scientific (USA); Tween 80—from Serva (Germany); DNA purification Kits—from Qiagen (Germany). The DNA manipulations were carried out according to the manufacturer’s instructions. All other reagents were of the highest purity grade and were purchased from domestic commercial suppliers (Russia).

### Synthesis of 3-methoxymethylated steroids

3-Hydroxy-∆^5(6)^-steroids (cholesterol, phytosterol, pregnenolone, DHEA) (25 mmol) were dissolved in 150 mL of formaldehyde dimethyl acetal in 500 mL flask furnished with calcium chloride lock. Then, 10 g of kieselguhr and 5 g of phosphorus pentoxide (by incremental portions) were added, and the reaction mixture was agitated within 6–8 h at a room temperature. The monitoring of dynamic reaction was carried out by TLC. After the reaction completion, the mixture was filtered off from insoluble matter, washed with 15 mL formaldehyde dimethylacetal, and the solvent was distilled under weak vacuum. Then, 50 mL of 5% (w/w) solution of sodium bicarbonate was added by portions and solid cake was triturated. The solid crude product was separated by filtration, rinsed with water to adjust pH to 7.0–7.2. The crude product was dissolved in 100 mL acetone under heating, filtered, and transferred to crystallizer (4 °C). After filtration, the mother solution was evaporated till 25 mL under vacuum. The solution was transferred to the fridge and after crystallization, filtration, and drying, the additional portion of product was obtained. The total yield of 3-methoxymethylated steroids was approximately 20–22 mmol, and their structures were characterized by HPLC, ^1^H- and ^13^C-NMR-spectroscopy (Table 1, Additional file 1: Fig. S7–S14).

**Table 1:** Characteristics of the obtained 3-methoxymethyl derivatives

Name and Chemical Structure	High-Performance Liquid Chromatography (HPLC), Mass-Spectrometry (MS), ^1^H- and ^13^C-Nuclear Magnetic Resonance Spectroscopy Data
	Retention time (Rt) 16.53 (mobile phases **I**); ^1^H-NMR (CDCl_3_) δ: 5.36 (m, 1H, 6-H), 4.69 (s, 2H, 3-OCH_2_OCH_3_), 3.42 (m, 1H, 3α-H), 3.37 (s, 3H, 3-OCH_2_OCH_3_), 1.01 (s, 3H, 19-CH_3_), 0.91 (d, *J* = 6.5 Hz, 3H, 21-CH_3_), 0.87 (d, *J* = 6.6 Hz, 3H, 26(27)-CH_3_), 0.86 (d, *J* = 6.6 Hz, 3H, 26(27)-CH_3_), 0.69 (s, 3H, 18-CH_3_). ^13^C-NMR (CDCl_3_) δ: 140.7 (C-5), 121.7 (C-6), 94.7 (3-OCH_2_OCH_3_), 76.9 (C-3), 56.7, 56.1, 55.2, 50.1, 42.3, 39.7, 39.5, 37.2, 36.7, 36.2, 35.8, 31.9, 31.8, 28.9, 28.2, 28.0, 24.3, 23.8, 22.8, 22.6, 21.0, 19.4, 18.7, 11.8
	Rt 14–17.5 (mobile phases **I**); ^1^H-NMR for a mixture of 3-methoxymethyl-sterols (CDCl_3_) δ: 5.36 (m, 1H, 6-H), 5.18–5.12 (m, 0.3H, 22(23)-H), 5.04–4.98 (m, 0.3H, 22(23)-H),4.69 (s, 2H, 3-OCH_2_OCH_3_), 3.43 (m, 1H, 3α-H), 3.37 (s, 3H, 3-OCH_2_OCH_3_), 1.03–0.69 (m, 25H, 19-CH_3_, 21-CH_3_, 24^2^-CH_3_, 24^1^-CH_3_, 26-CH_3_, 27-CH_3_, 18-CH_3_). ^13^C-NMR for a mixture of 3-methoxymethyl-sterols (CDCl_3_) δ: 140.7 (C-5), 138.3 (C-22), 129.2 (C-23), 121.7 (C-6), 94.6 (3-OCH_2_OCH_3_), 76.9 (C-3), 56.8, 56.7, 56.0, 55.9, 55.1, 51.2, 50.1, 45.8, 42.3, 42.2, 40.5, 39.7, 39.6, 39.5, 37.2, 36.7, 36.1, 35.9, 33.9, 33.7, 32.4, 31.9, 31.9, 31.4, 30.2, 29.1, 28.9, 28.2, 26.0, 25.4, 24.3, 24.3, 23.0, 21.2, 21.1, 21.0, 20.2, 19.8, 19.3, 19.0, 18.8, 18.7, 18.2, 15.4, 15.3, 12.2, 12.0, 12.0, 11.8
	Rt 3.77 (mobile phases **I**); ^1^H-NMR (CDCl_3_) δ: 5.36 (m, 1H, 6-H), 4.69 (s, 2H, 3-OCH_2_OCH_3_), 3.43 (m, 1H, 3α-H), 3.37 (s, 3H, 3-OCH_2_OCH_3_), 2.53 (m, 1H. 17α-H), 2.13 (s, 3H, 21-CH_3_), 1.01 (s, 3H, 19-CH_3_), 0.63 (s, 3H, 18-CH_3_). ^13^C-NMR (CDCl_3_) δ: 209.5 (C-20), 140.7 (C-5), 121.4 (C-6), 94.6 (3-OCH_2_OCH_3_), 76.8 (C-3), 63.7, 56.8, 55.1, 50.0, 43.9, 39.5, 38.8, 37.2, 36.7, 31.8, 31.7, 31.5, 28.8, 24.4, 22.7, 21.0, 19.3, 13.2
	Rt 3.33 (mobile phases **I**); ^1^H-NMR (CDCl_3_) δ: 5.39 (m, 1H, 6-H), 4.69 (s, 2H, 3-OCH_2_OCH_3_), 3.43 (m, 1H, 3α-H), 3.38 (s, 3H, 3-OCH_2_OCH_3_), 1.04 (s, 3H, 19-CH_3_), 0.89 (s, 3H, 18-CH_3_). ^13^C-NMR (CDCl_3_) δ: 221.1 (C-17), 140.9 (C-5), 120.9 (C-6), 94.7 (3-OCH_2_OCH_3_), 76.7 (C-3), 55.2, 51.7, 50.2, 47.5, 39.5, 37.1, 36.8, 35.8, 31.4, 31.3, 30.7, 28.8, 21.8, 20.3, 19.4, 13.5

### Bacterial strains and plasmids

A strain of *Mycolicibacterium smegmatis* mc^2^ 155 was kindly provided by Dr. E. Noens (European Molecular Biology Laboratory (EMBL), Hamburg Outstation, Hamburg, Germany). The *M. smegmatis* cultures were maintained on M3 medium [28] at 37 °C. *E. coli* strain DH5α (Thermo Fisher Scientific, USA) were grown at 37 °C in LB medium [37]. Solid media were prepared with bacto agar at a final concentration of 1.5% (w/v). *E. coli*-mycobacteria shuttle expression vector pMyNT [29] with inducible acetamidase promoter is a gift by Dr. A. Geerlof (EMBL, Hamburg). Hygromycin B was used to select recombinant strains and to maintain the plasmid pMyNT. Plasmid pTrc99A/mP450scc [38] was kindly provided by Prof. M.R. Waterman (University of Texas, Southwestern Medical Center, Dallas, TX, USA). The pBar_Triple plasmid was constructed earlier [14]. Ampicillin (100 mg/L) was used to maintain the plasmids pTrc99A/mP450scc and pBar_Triple in the *E. coli* strain DH5α.

### Construction of plasmids

Plasmids pTrc99A/P450scc [38] containing cDNA for mature bovine cytochrome P450scc and the pBar_Triple [14] containing cDNA encoding three cholesterol hydroxylase/C20-C22 lyase system (CH/L) proteins (AdR, Adx, and P450scc) were used. Molecular cloning work was performed by using standard protocols [39]. 732 bp *Nco*I-*Bgl*II and 840 bp *Bgl*II-*Hin*dIII fragments from pTrc99A/mP450scc [38] plasmid were ligated with *Nco*I-*Hind*III digested mycobacterial vector pMyNT resulting plasmid pNS10. cDNA for fragment of tricystronic AdR-Adx-P450scc cassette of pBar_Triple plasmid was amplified by PCR using primers Pre5N1 (5′-TTCCAGGGCGCCATGGCGAGCACTCAAGAACAAACTCCCCAG-3′) and Pre3H3 (5′-CGACATCGATAAGCTTTCACGCCTGGGGCGGGTCCTGGTT-3′). Additional GCG/Ala codon was added on the N-terminus of the AdR to introduce *Nco*I site in the Pre5N1 primer (underlined). The pMyNT vector linearized by *Nco*I-*Hin*dIII digestion and 3.3 kb PCR amplified fragment with cDNA, encoding RBS-AdR-RBS-Adx-RBS-P450scc were transferred to *E. coli* DH5α competent cells for homologous recombination of the vector and the PCR-fragment generating plasmid pNS11. The sequence of the cloned 3.3 kb cassette with three cDNAs and junctions with vector was validated by Sanger sequencing.

### Protein expression in *M. smegmatis* mc^2^ 155

Competent *M. smegmatis* mc^2^ 155 cells were transformed with plasmid DNA of pMyNT, pNS10 or pNS11 by electroporation [40]. The transformants were selected on agar M3 medium with the addition of hygromycin B (75 mg/L).

In order to express the recombinant proteins, the cells of individual colonies were grown in 50 mL of medium M3 containing hygromycin B (50 mg/L) aerobically at 37 °C and 220 rpm up to OD_600_ = 0.8. Synthesis of recombinant proteins was induced by an addition of acetamide (2 g/L), and then bacteria were cultivated at 32 °C and 220 rpm. The samples (1 mL) of cultures were taken repeatedly within two days; bacterial cells were harvested, re-suspended in sample buffer [41] and heated at 100 °C within 10 min. Protein synthesis analysis was performed by denaturing 10% or 15% polyacrylamide gel electrophoresis (SDS-PAGE) [41] and Western blotting using primary IgG antibodies against bovine P450scc, AdR, and Adx [14].

### In Vivo Activity of cholesterol hydroxylase/C20-C22 lyase system (CH/L)

#### Cultivation of recombinant strain *M. smegmatis* mc^2^ 155

The recombinant *M. smegmatis* mc^2^ 155 strain was grown in two stages in 750 mL Erlenmeyer flasks with M3 medium (50 mL) supplemented with fructose (10 g/L) and hygromycin B (50 mg/L) on a Multitron Standard shaker-incubator (Infors HT, Switzerland) at 37 °C and 200 rpm. At the first stage, the strain was grown within 40–48 h. The obtained seed culture (10%, v/v) was inoculated into a similar medium and cultivated within 24 h (the second stage) under the same conditions.

#### Bioconversion of sterols and their 3-methoxymethyl derivatives

Bioconversion of sterols (cholesterol, phytosterol) and their 3-methoxymethyl derivatives (MOM-cholesterol, MOM-phytosterol) (2.33 mM) by recombinant strain (10%, v/v, of the second stage culture) was carried out in 250 mL flasks with of M3 medium (15 mL), supplemented with 0.4 mL of fructose solution (0.5 g/mL) and 1 mL of 0.5 M K-phosphate buffer (pH 6.8). After 12 h of growth, acetamide was added to a final concentration of 2 g/L. After 24 h of growth, 1.2 mL of MCD solution (in a molar ratio to substrate of 5:1), 0.4 mL of fructose solution (0.5 g/mL), and the substrate in a hot 2-propanol solution were added. The alcohol concentration did not exceed 1.25% (v/v). Then, fructose was added daily to a final concentration of 10 g/L.

Optimization of acetamide induction conditions was carried out using a MOM-cholesterol as a substrate (1.0 g/L, corresponds to 2.33 mM). The strain growth and bioconversion of substrates was carried out on a Multitron Standard shaker-incubator (Infors HT, Switzerland) at 37 °C and 200 rpm. The experiments were carried out in three replicates.

### Pregnenolone production and isolation

#### Isolation of MOM-pregnenolone from culture medium

The bioconversion of MOM-cholesterol by *M. smegmatis* mc^2^ 155 pNS11 strain to MOM-pregnenolone was performed in 750 mL flasks as described in paragraph “Bioconversion of sterols and their 3-methoxymethyl derivatives”. The volumes of the medium, solutions and inoculum were increased by 2.5 times, correspondently. The expression of heterologous genes was initiated by adding acetamide (2 g/L) to the bioconversion medium at 0, 12, 24, and 36 h of growth.

Upon completion of MOM-cholesterol bioconversion with *M. smegmatis* mc^2^ 155 pNS11, the biomass was separated by centrifugation of the culture broth (1000 mL) at 5,600 × *g* at 25 °C within 1 h. The supernatant was extracted successively with three portions of ethyl acetate with a total volume of 1.5 L with phase separation in a chemical reactor equipped with a stirrer within 30–40 min. The combined organic phase containing the target steroid was evaporated under a vacuum (0.1 atm), 50 °C to a volume of 300 mL, and it was washed three times with distilled water (50 mL) and was dried over anhydrous sodium sulfate (30 g) within a day. Then organic phase was evaporated on rotary evaporator at 0.1 atm, 50 °C till termination of solvent distillation. After that 50 mL of hexane was added and oily residue was washed under normal pressure till initiation of copious crystallization. Crystallization was completed at 4 °C. The crystals were recovered by filtration, washed with 10 mL of cold hexane and dried to give 0.8 g of crude crystalline steroid product. The crude product was re-crystallized from 4 mL of acetone. Then the crystals were dried during 3 h till constant weight in vacuum drying oven at 50 °C under reduced pressure.

#### Acid hydrolysis of MOM-pregnenolone

MOM-pregnenolone (0.57 g) was heated within 1 h under reflux with 10 mL methanol, 2 mL water, and 1 mL concentrated hydrochloric acid. Then the mixture was cooled, and suspension obtained poured in 70 mL of ice water, and filtered off, thus obtaining the crude product. The crude product was re-crystallized from 15 mL of organic mixture heptane:benzene (1:1, v/v). Product was dried till constant weight in vacuum drying oven at 50 °C.

#### Steroid metabolite isolation

Steroids were extracted from the supernatant (~ 150 mL; 8000 × g, 30 min) with ethyl acetate (100 mL) three times and the pooled organic extract was concentrated on a rotary evaporator. The separation of steroids was carried out as described earlier [33] by using System A (benzene:acetone, 40:10, v/v) or System B (n-heptane:acetone:methanol (30:25:0.5, v/v/v).

### Steroid analyses

#### Thin layer chromatography (TLC)

The samples of cultivation broth (0.25 mL) were taken and extracted with 0.5 mL of ethyl acetate. The organic extracts were applied to TLC plates (ALUGRAM SIL G/UV254, Macherey–Nagel, Düren, Germany) and developed in System A (benzene:acetone, 40:10, v/v; substrates: cholesterol, phytosterol and their bioconversion derivatives) and in System B (n-heptane:acetone:methanol (30:25:0.5, v/v/v; substrates: MOM-cholesterol, MOM-phytosterol and their bioconversion derivatives). Steroids with 3-oxo-4-ene moiety were visualized as described earlier [33]. To detect steroids with 3β-hydroxy-5-ene moiety and substituted at the 3rd position in ring A, the TLC plates were treated with a MnCl_2_ solution [42] and heating at 105 °C within 5–10 min and visualized under UV light (365 nm).

#### High-performance liquid chromatography (HPLC)

Sample preparation: an aliquot of the culture liquid (0.25 mL) was diluted 10 times with a mixture of acetonitrile:isopropanol (50:45, v/v), mixed and centrifuged within 8 min at 12,100 × g. The supernatant was used for analysis. HPLC analyses were performed on an Agilent Infinity 1200 system (Agilent Technologies, Germany) with a Symmetry C18 column (4.6 × 250 mm, 5 μm) with a Symmetry C18 precolumn (3.9 × 20 mm, 5 μm,) (Waters, Milford, MA, USA) at 50 °C and a flow rate of 1 mL/min. Steroid assay was performed using two mobile phases: **I**—acetonitrile:2-propanol:water (50:45:5 (v/v/v)) and **II**—acetonitrile:water:TFA (60:40:0.02 (v/v/v)) with gradient elution as described [43], UV-detection at 200 (**I**) and 240 nm (**II**). Calibrations were performed by the external standard method based on peak areas. The results were processed using the ChemStation Rev. software. B.04.03 (Agilent Technologies, USA). Retention times (Rt) for mobile phase **I**: cholesterol, Rt 10.3; phytosterols, Rt 9.5–10; pregnenolone, Rt 3.16; for mobile phase **II**: progesterone, Rt 10.56. Rt of 3-methoxymethyl derivatives are given in Table 1.

#### Mass-Spectrometry (MS), ^1^H-NMR (Nuclear Magnetic Resonance) and ^13^C-NMR Spectroscopy

MS spectra of compounds were recorded on a tandem mass spectrometer Thermo Finnigan LCQ Advantage MAX (USA) in the positive ion [M + H^+^] mode at an evaporator temperature of 200 °C and capillary temperature of 180 °C. MS/MS spectra were obtained using normalized collision energy (Normolized Collision EnergyTM) ranging from 20 to 40%. Data were collected and processed using the Xcalibur software.

^1^H- and ^13^C-NMR spectra were recorded at 400 and 100.6 MHz respectively with a Bruker Avance 400 spectrometer. Chemical shifts were measured relative to tetramethylsilane and solvent signal. Only the characteristic signals in the ^1^H- and ^13^C-NMR spectra of 3-methoxymethyl derivatives are shown in Table 1.

### Supplementary Information

**Additional file 1:** Additional Figures S1–S14.

## Data Availability

The data that support the findings of this study are available from the corresponding author upon reasonable request.
