# Supplementary material for: Pregnenolone and progesterone production from natural sterols using recombinant strain of Mycolicibacterium smegmatis mc2 155 expressing mammalian steroidogenesis system
Source: Microb Cell Fact. 2024 Apr 9;23:105. doi: 10.1186/s12934-024-02385-2 (PMC11005228; doi:10.1186/s12934-024-02385-2)
Supplement: Supplementary file 1 — Additional file 1: Additional Figures S1–S14. [file 12934_2024_2385_MOESM1_ESM.pdf]

Supplementary Information

Microbial Cell Factories

Pregnenolone and progesterone production from natural sterols using recombinant strain of *Mycolicibacterium smegmatis* mc^2 155 expressing mammalian steroidogenesis system

Karpov M. V.[1*], Strizhov N. I.[1], Novikova L.A.[2], Lobastova T. G.[1], Khomutov S. M.[1],
Shutov A. A.[1], Kazantsev A.V.[3], Donova M. V.[1*]

[1] Laboratory of Bioengineering of Microbial Producers, G.K. Skryabin Institute of Biochemistry and Physiology of Microorganisms, RAS, Federal Research Center "Pushchino Scientific Center for Biological Research of the Russian Academy of Sciences", 142290 Pushchino, Russia

[2]Belozersky Institute of Physico-Chemical Biology, Lomonosov Moscow State University, Leninskie Gory 1/40, 119234 Moscow, Russia

[3]Chemistry Department, Lomonosov Moscow State University, Leninskie Gory 1/3, 119991 Moscow, Russia

* Correspondence:

Mikhail Karpov

mikhail.v.karpov@mail.ru

Marina Donova

donova@ibpm.pushchino.ru

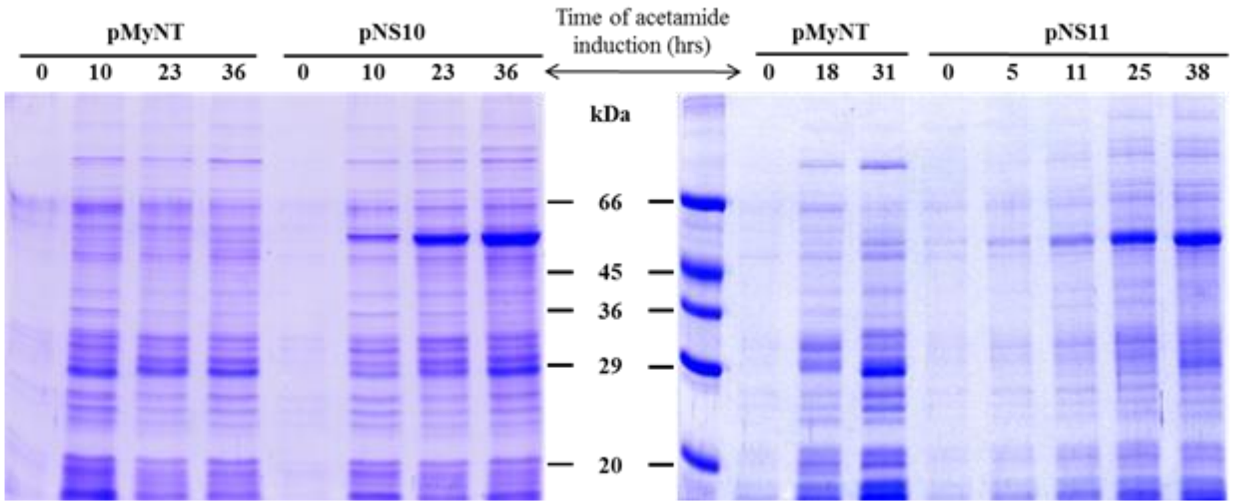

Fig. S1 SDS-PAGE analysis of proteins synthesized by acetamide induced *M. smegmatis* mc^2 155 cells carrying recombinant plasmids

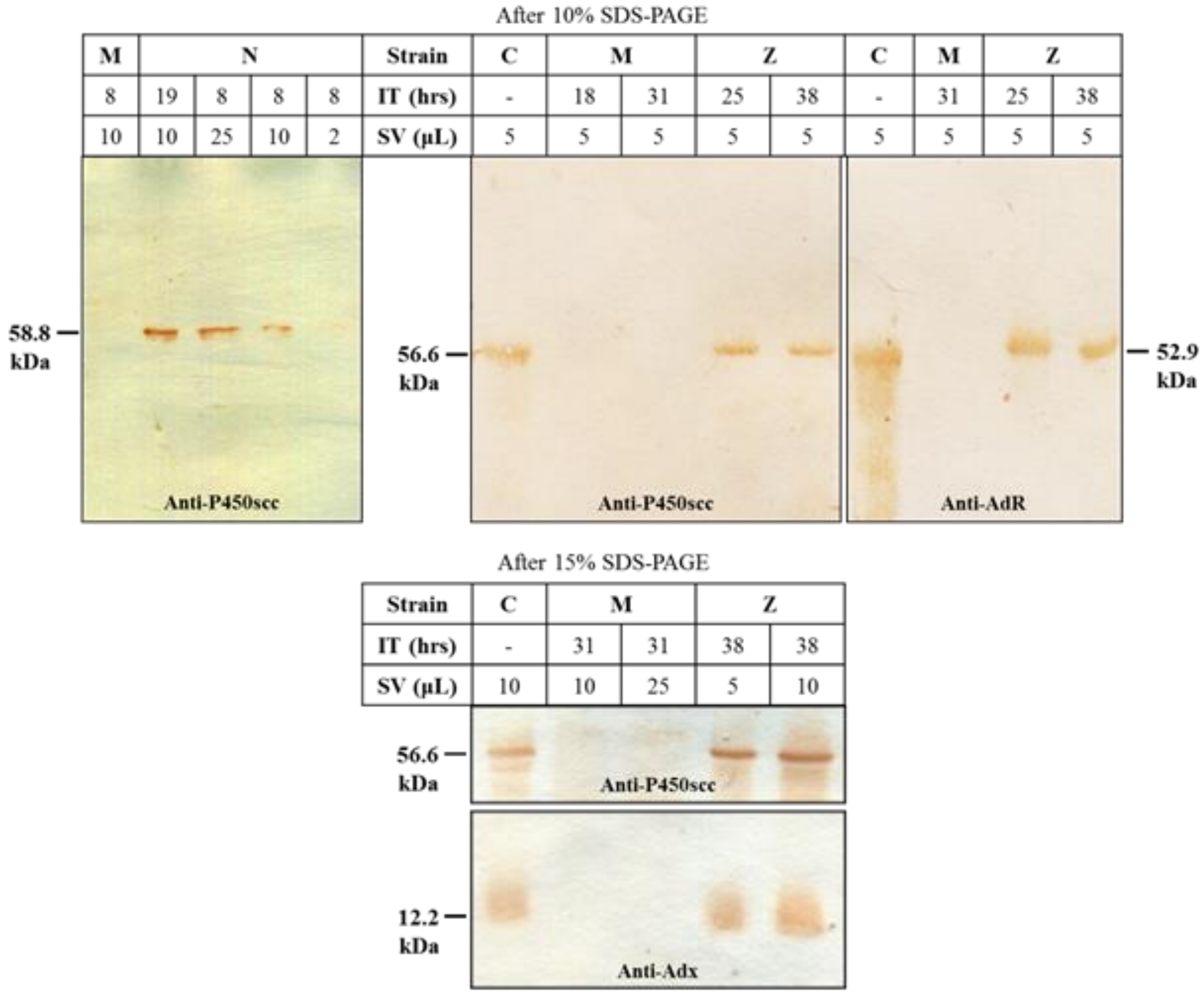

Fig. S2 Western immunoblotting of proteins synthesized by acetamide induced *M. smegmatis* mc^2 155 cells carrying pMyNT (**M**) (negative control), pNS10 (**N**) and pNS11 (**Z**) plasmids. Antibodies against bovine P450scc, AdR or Adx were used. The protein preparation of IPTG-induced *E. coli* DH5αc/ pBar_Triple (Makeeva et al., 2013) was used as a positive control (**C**). The acetamide induction time (**IT**, hours) and volumes of samples applied to the gel (**SV**, μL) are presented in the figure

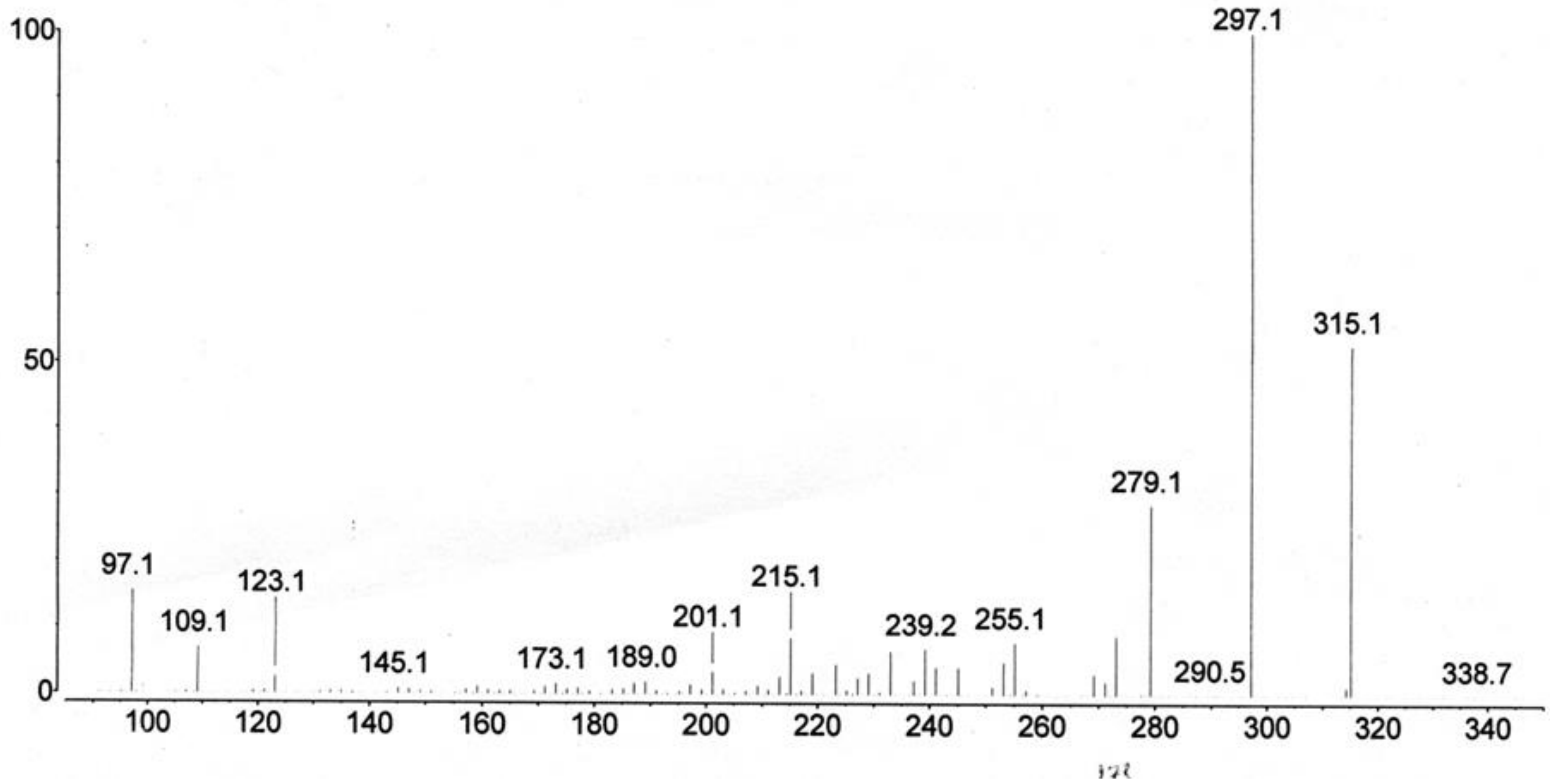

Fig. S3 Mass spectrum [M+H]⁺ of progesterone (substrate-cholesterol)

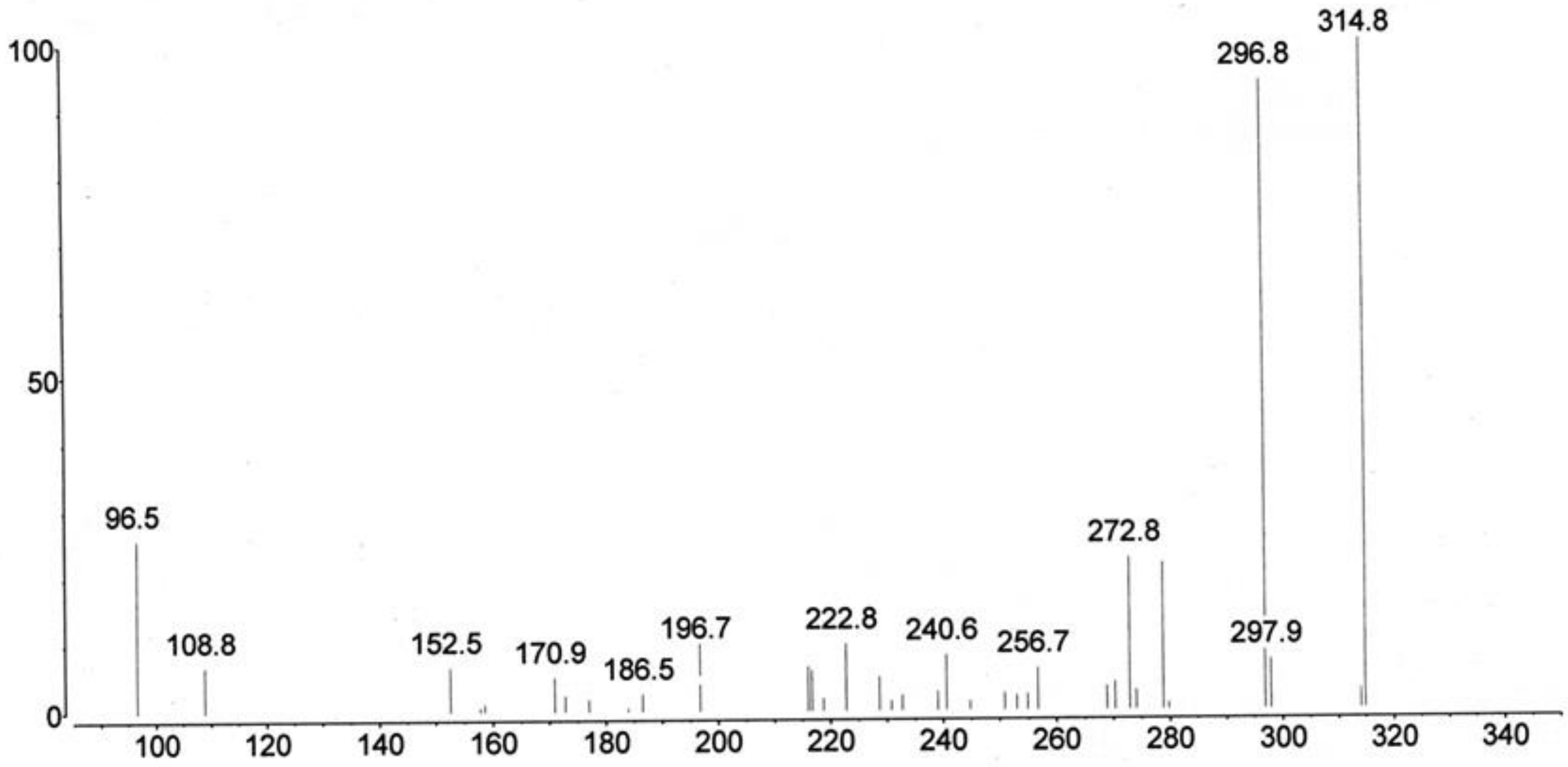

Fig. S4 Mass spectrum $[M+H]^+$ of progesterone (substrate-phytosterol)

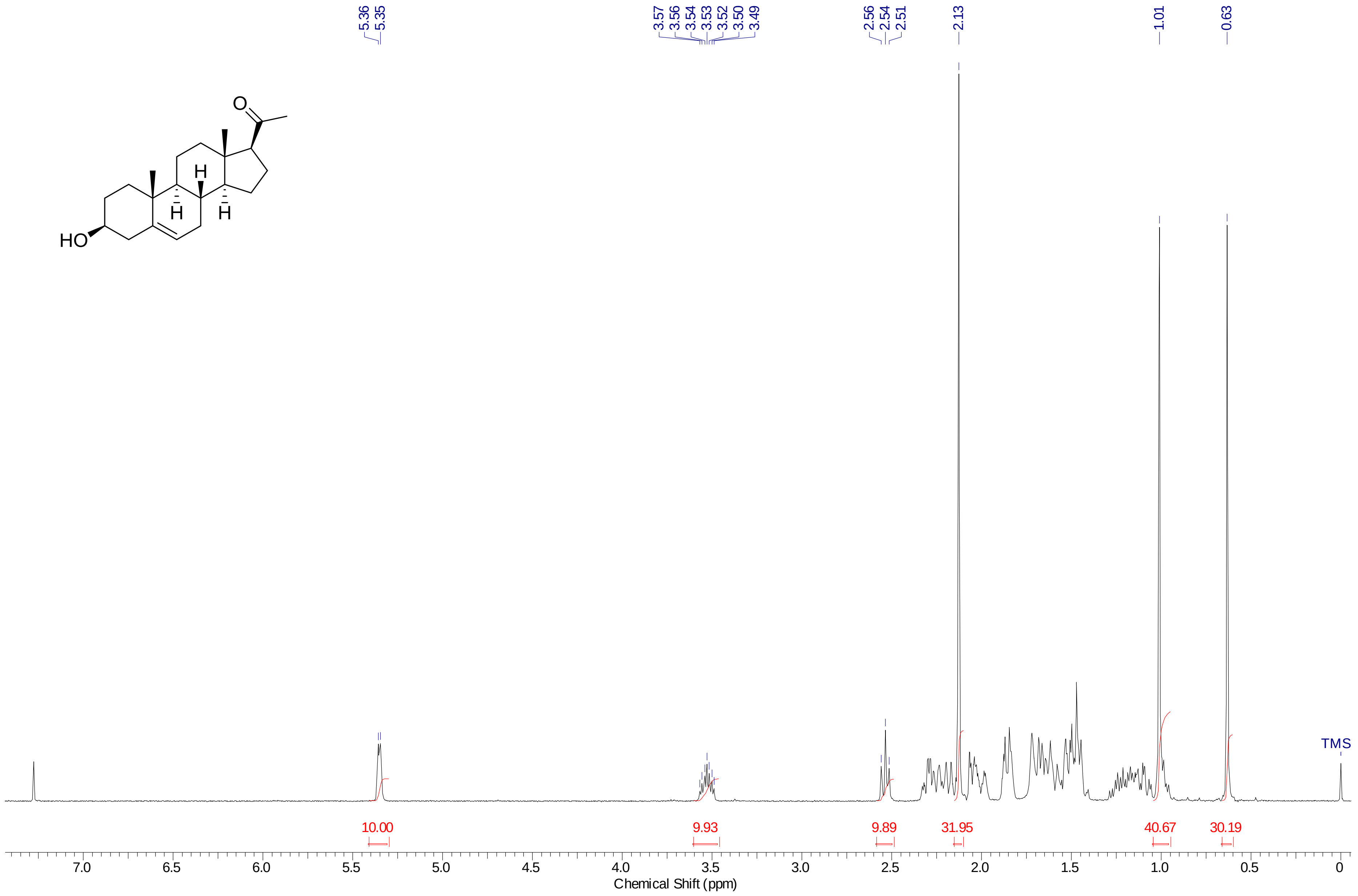

Fig. S5 ^{1}H -NMR spectrum of pregnenolone

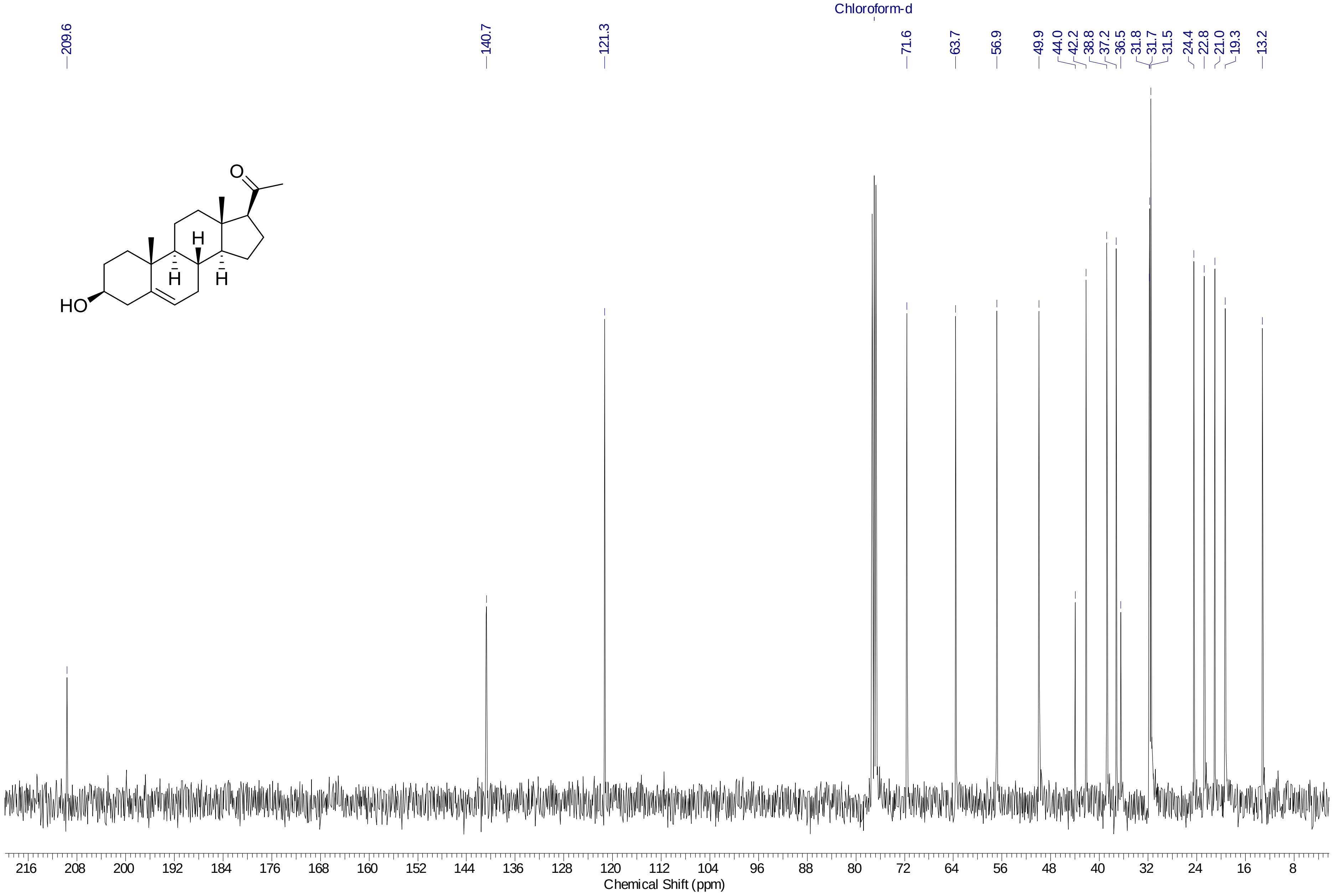

Fig. S6 ^{13}C-NMR spectrum of pregnenolone

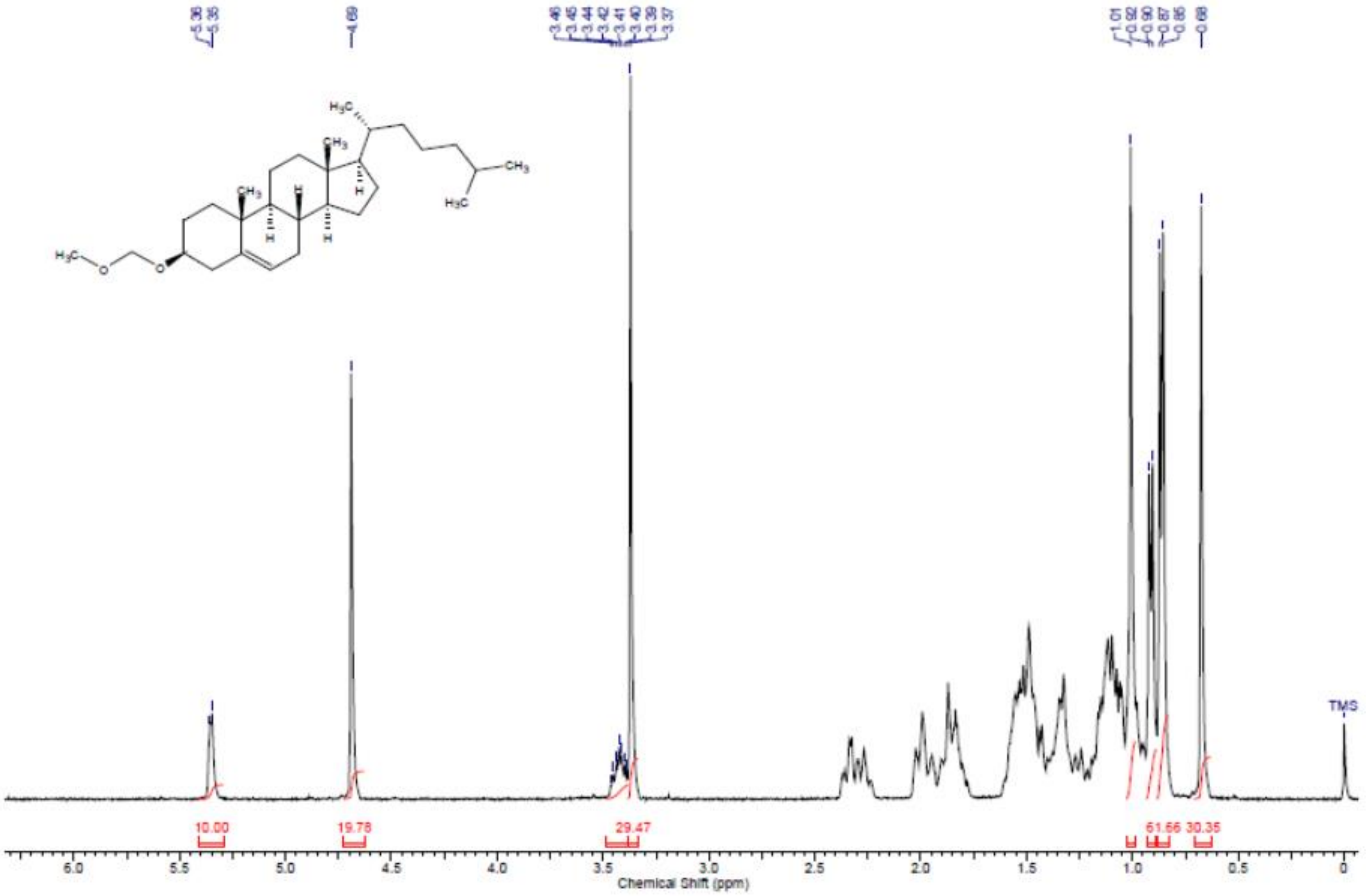

Fig. S7 ^{1}H-NMR spectrum of MOM-cholesterol

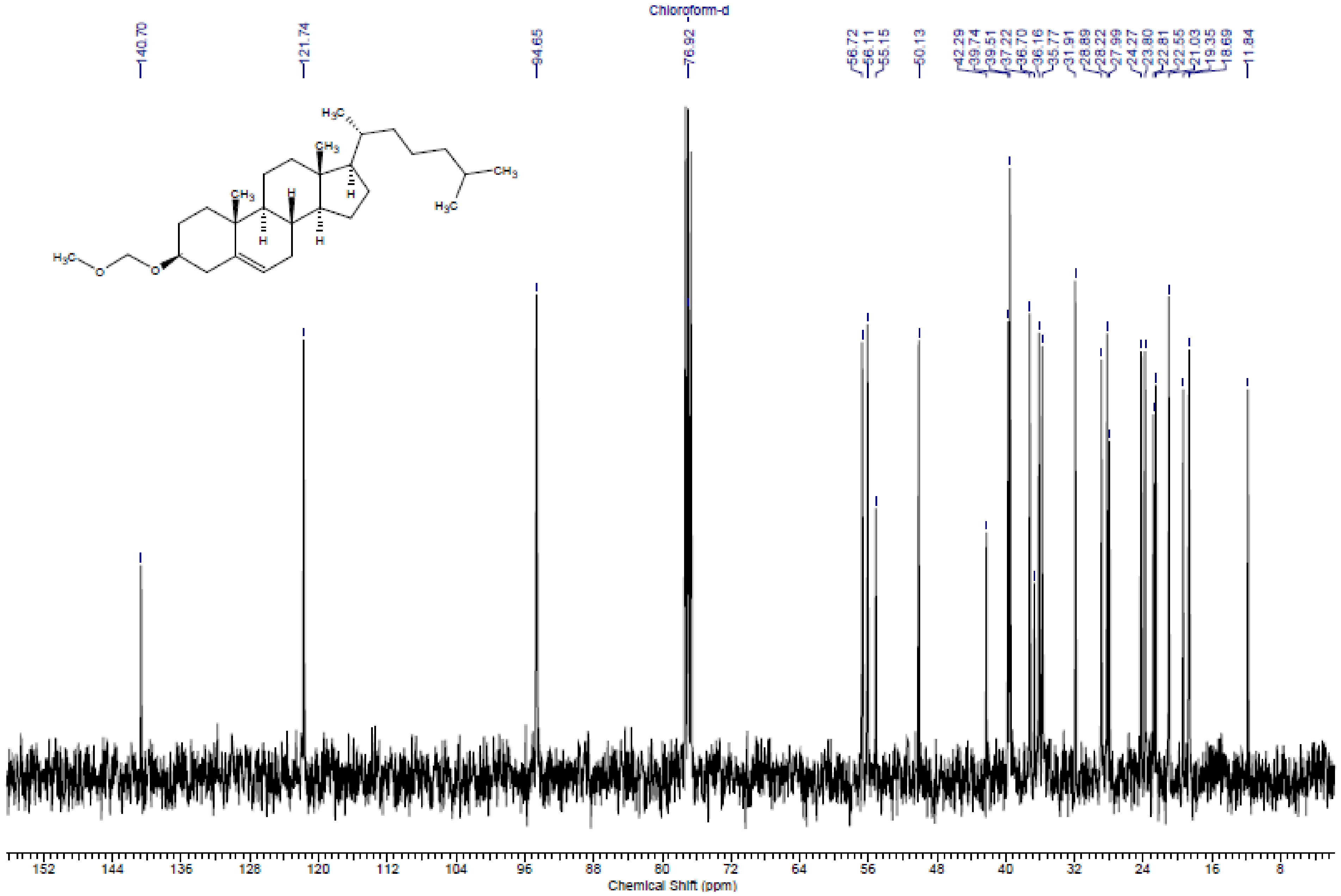

Fig. S8 ^{13}C-NMR spectrum of MOM-cholesterol

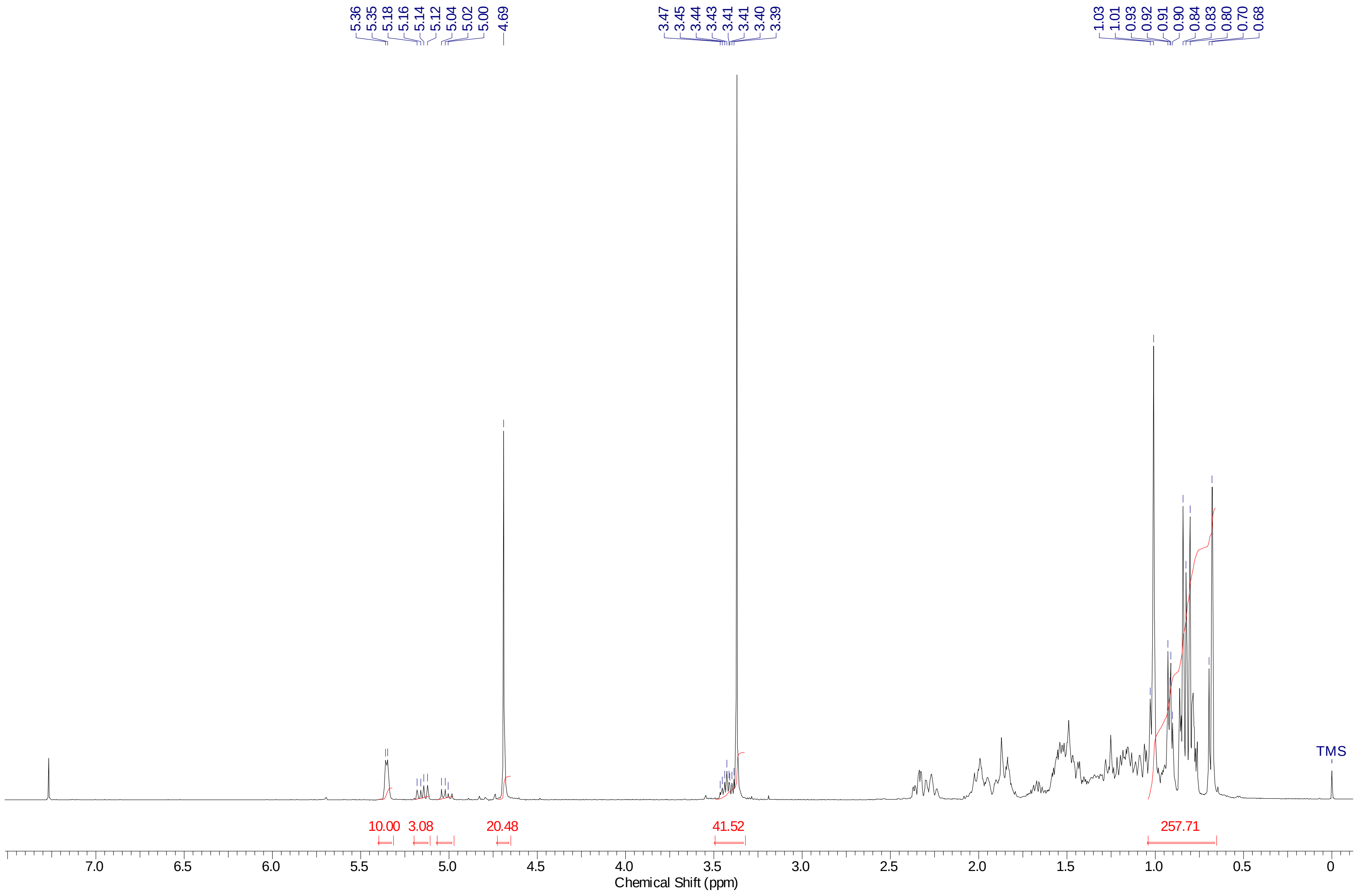

Fig. S9 ^{1}H-NMR spectrum of MOM-phytosterol

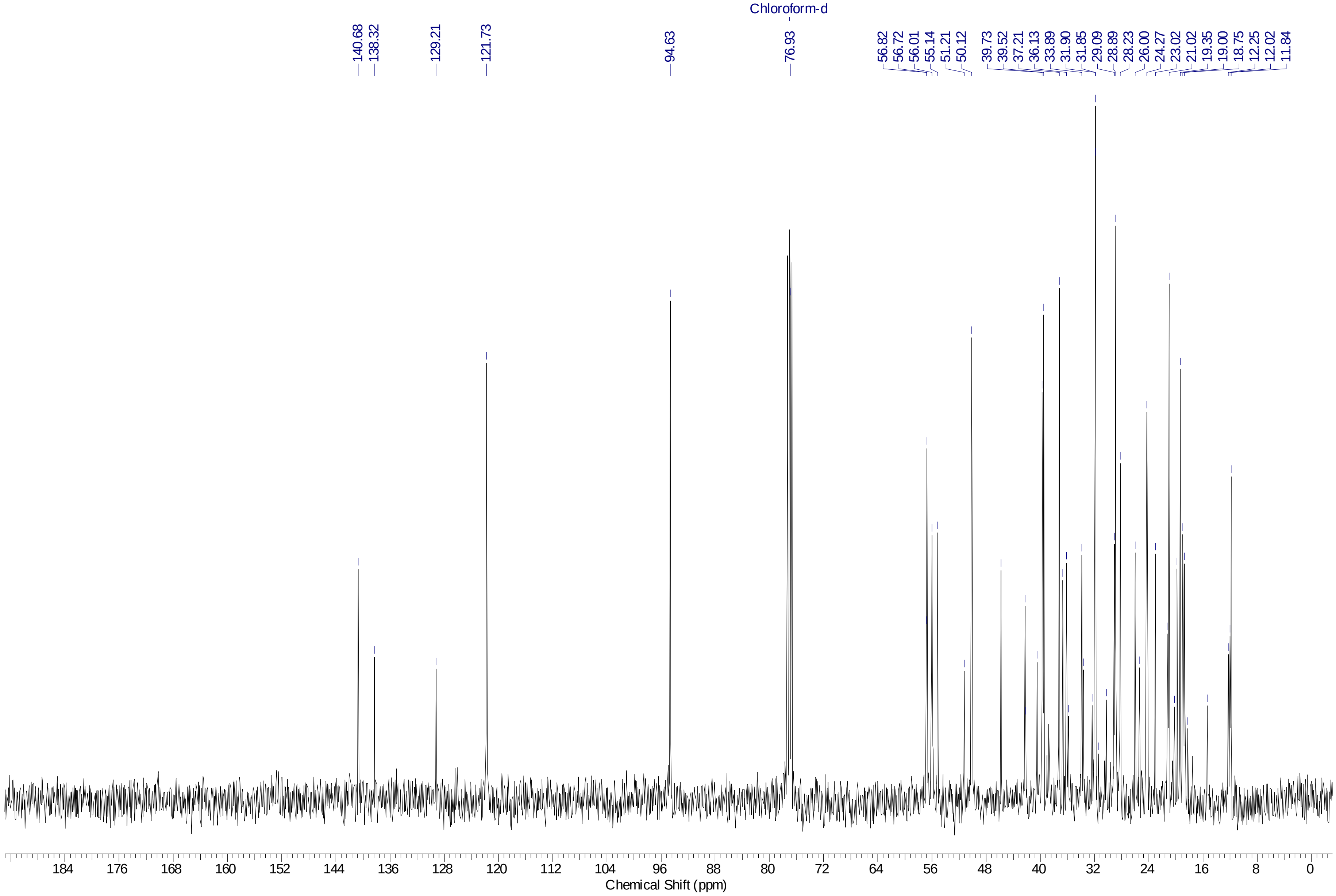

Fig. S10 ^{13}C-NMR spectrum of MOM-phytosterol

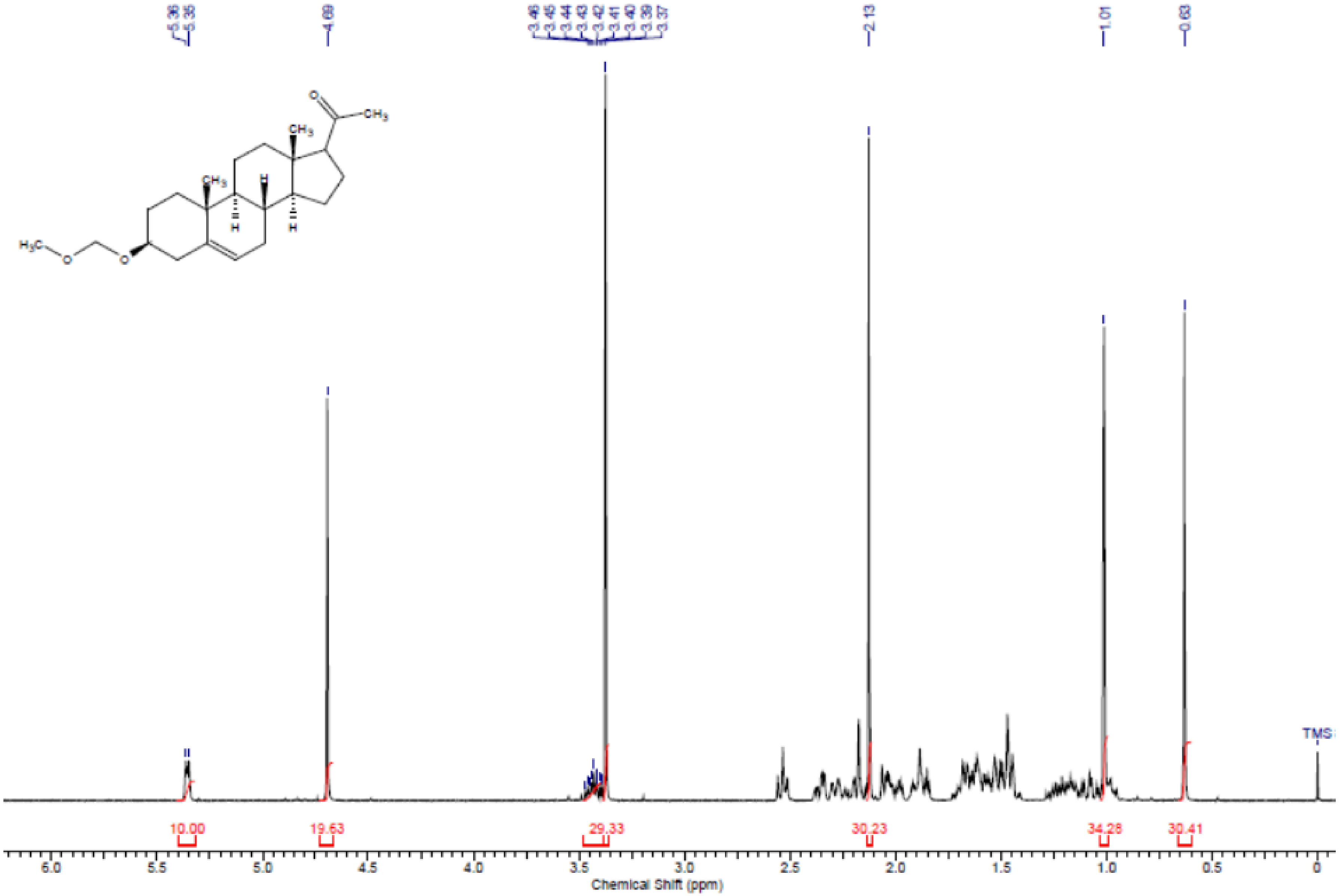

Fig. S11 ^{1}H-NMR spectrum of MOM-pregnenolone

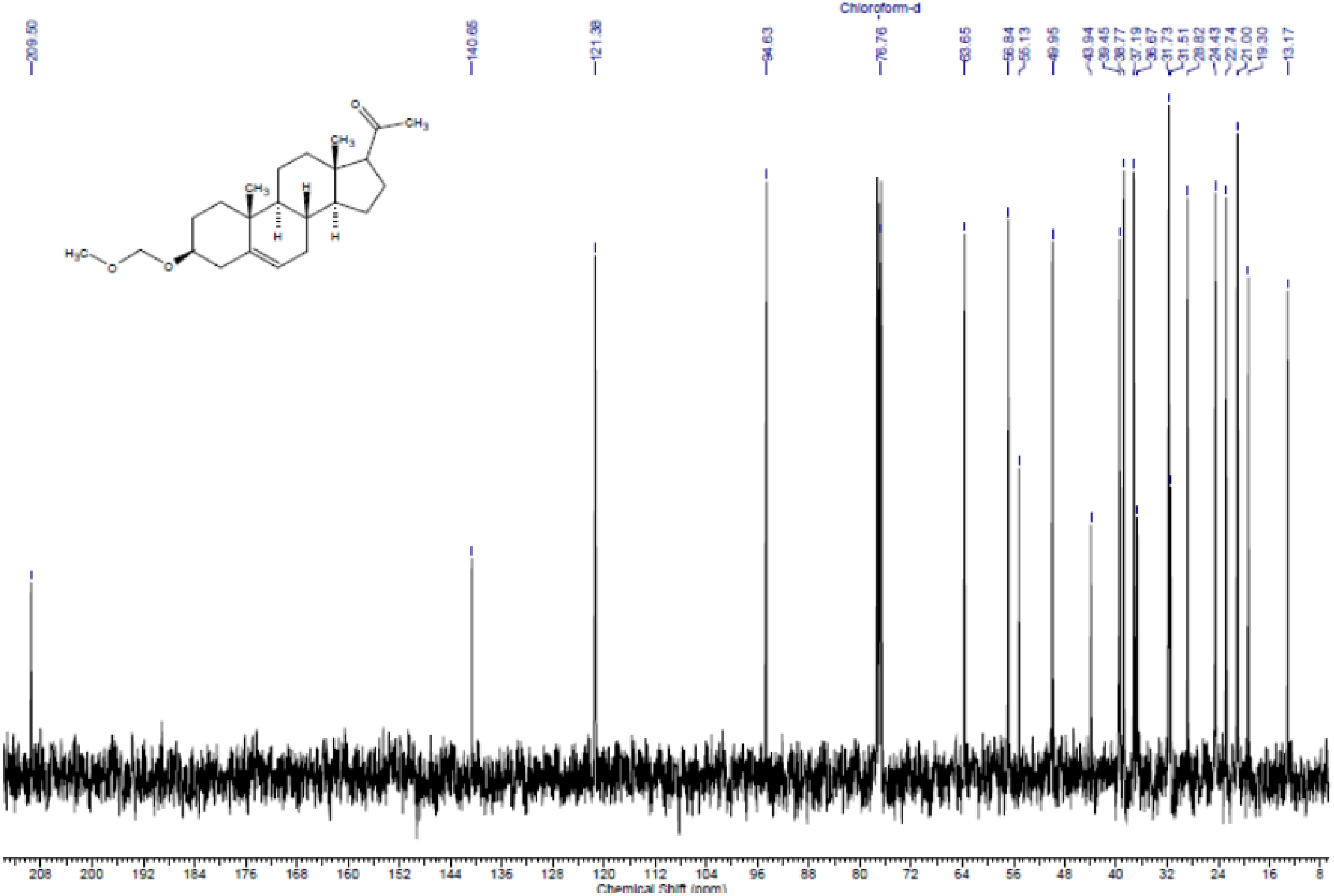

Fig. S12 ^{13}C-NMR spectrum of MOM-pregnenolone

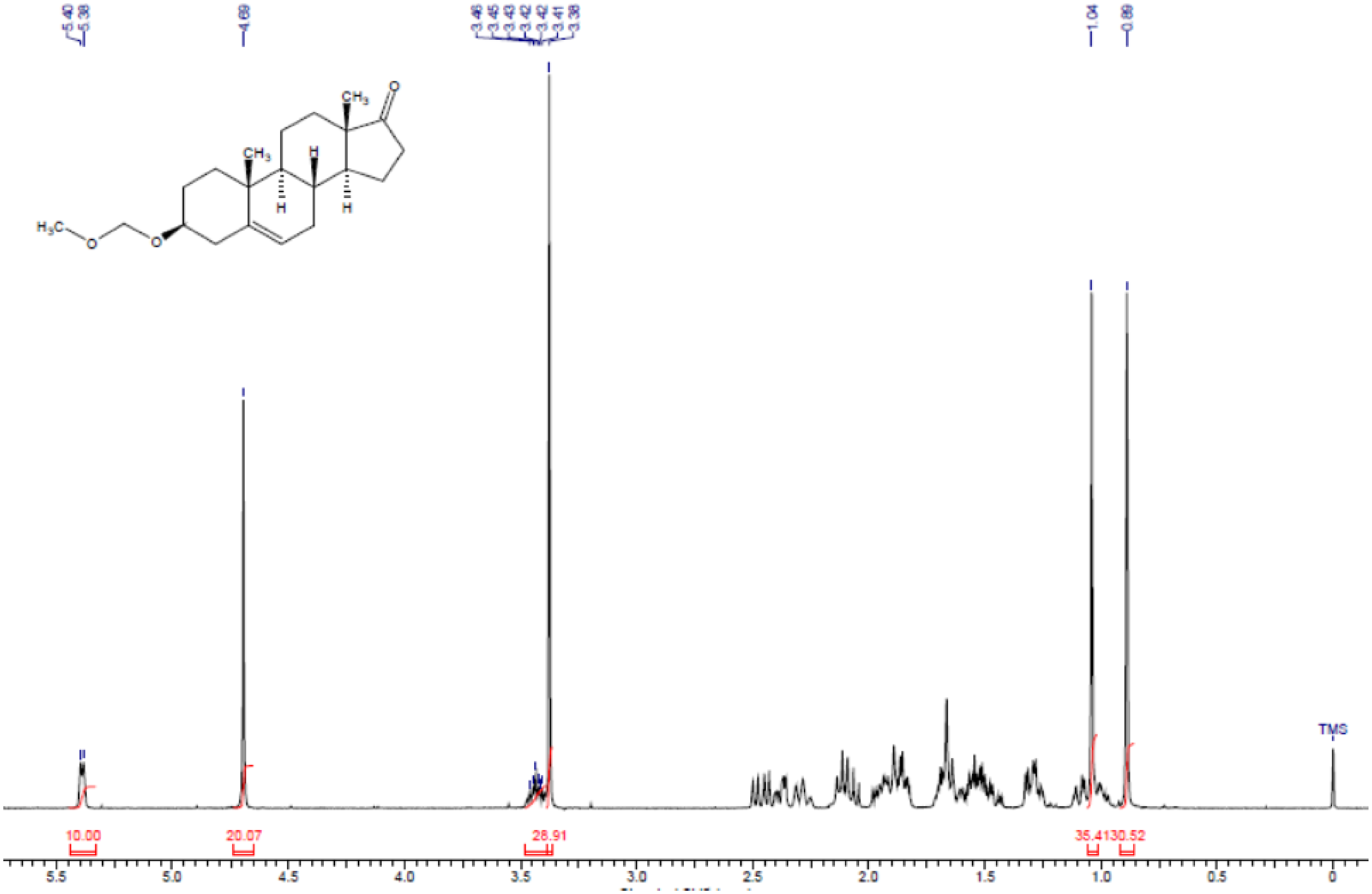

Fig. S13 ^{1}H -NMR spectrum of MOM-DHEA

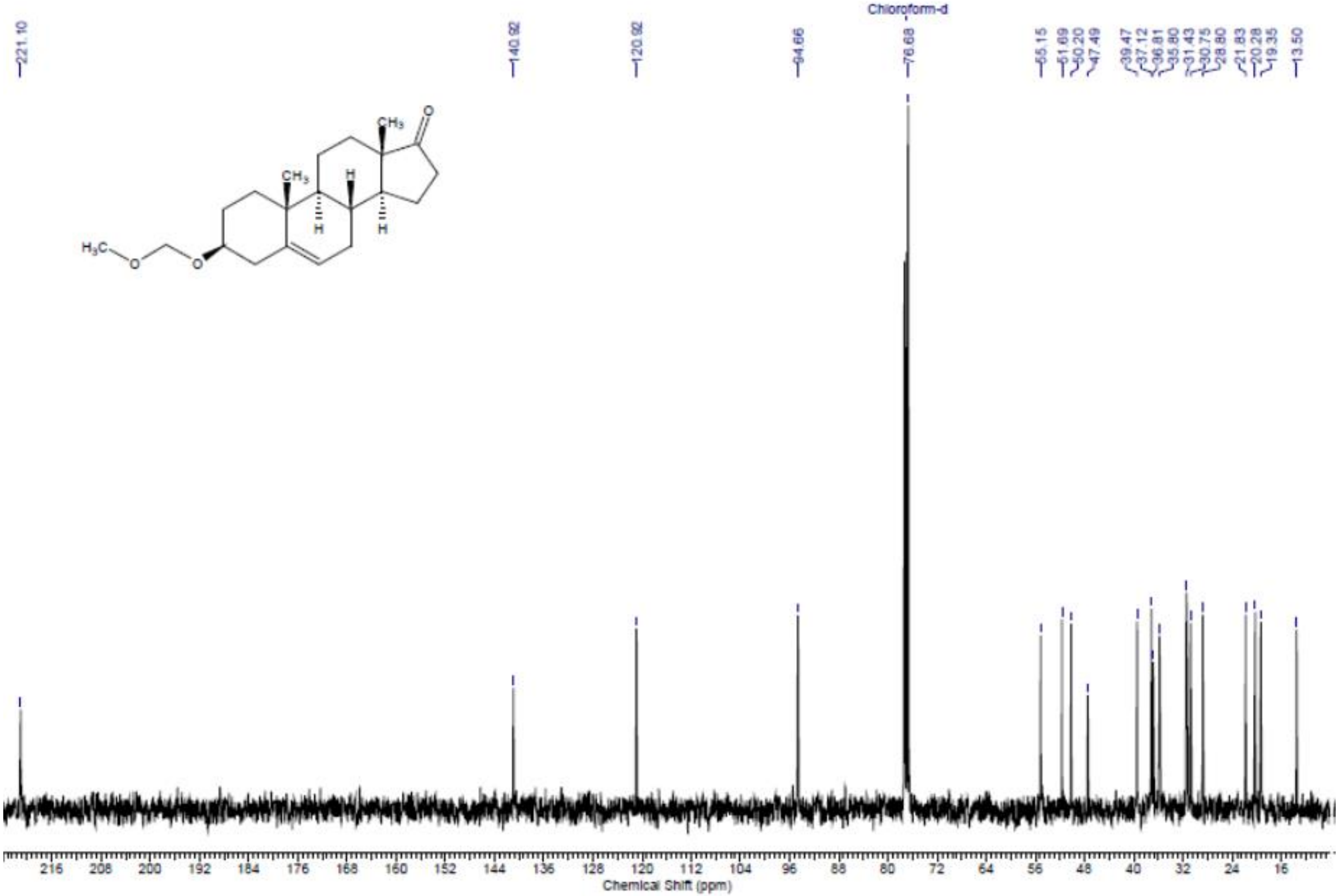

Fig. S14 ^{13}C-NMR spectrum of MOM-DHEA
